# XAF1 antagonizes TRIM28 activity through the assembly of a ZNF313-mediated destruction complex to suppress tumor malignancy

**DOI:** 10.1186/s43556-024-00224-9

**Published:** 2024-11-13

**Authors:** Seung-Hun Jang, Hwi-Wan Choi, Jieun Ahn, Sungchan Jang, Ji-Hye Yoon, Min-Goo Lee, Sung-Gil Chi

**Affiliations:** Department of Life Sciences, Korea University, Seoul, 02841 Republic of Korea

**Keywords:** XAF1, TRIM28, ZNF313, Ubiquitination, Tumor suppression

## Abstract

X-linked inhibitor of apoptosis-associated factor 1 (XAF1) is a stress-inducible pro-apoptotic protein that is commonly inactivated in multiple human cancers. Nevertheless, the molecular basis for its tumor suppression function remains largely uncharacterized. Here we report that XAF1 antagonizes the oncogenic activity of tripartite motif containing 28 (TRIM28) ubiquitin E3 ligase through zinc finger protein 313 (ZNF313)-induced ubiquitination and proteasomal degradation. XAF1 exerts apoptosis-promoting effect more strongly in *TRIM28*^+*/*+^ versus *XAF1*^*−/−*^ tumor cells and suppresses tumor cell growth, migration, invasion, and epithelial-to-mesenchymal transition and xenograft tumor growth in a highly TRIM28-dependent fashion. Mechanistically, XAF1 interacts directly with the RING domains of TRIM28 and ZNF313 through the ZF6 and ZF7 domain, respectively, thereby facilitating ZNF313 interaction with and ubiquitination of TRIM28. A mutant XAF1 lacking either ZF6 or ZF7 domain exhibits no activity to promote TRIM28 ubiquitination. By destabilizing TRIM28, XAF1 blocks TRIM28-driven ubiquitination of p53 and RLIM, p53-HDAC1 interaction, and TWIST1 stabilization. Intriguingly, TRIM28 destabilizes XAF1 through K48-linked polyubiquitination and proteasomal degradation to protect tumor cells from apoptotic stress, indicating its role as an intrinsic antagonist against XAF1 and the antagonistic interplay of XAF1 and TRIM28. XAF1 expression is inversely correlated with TRIM28 expression in cancer cell lines and tumor tissues and more tightly associated with the survival of TRIM28-high versus TRIM28-low patients. Together, this study uncovers a novel mechanism by which XAF1 suppresses tumor malignancy and an important role for XAF1-TRIM28 interplay in governing stress response, illuminating the mechanistic consequence of its alteration during tumorigenic process.

**Supplementary Information:**

The online version contains supplementary material available at 10.1186/s43556-024-00224-9.

## Introduction

X-linked inhibitor of apoptosis (XIAP)-associated factor 1 (XAF1) is originally reported as a pro-apoptotic protein that interacts with XIAP to antagonize its anti-apoptotic activity [1, 2]. Epigenetic inactivation of *XAF1* is observed in various types of human cell lines and primary tumor tissues and associated with the malignant progression of multiple tumors [3–5]. In addition to apoptosis-promoting role, XAF1 is involved in various cellular aspects, including autophagy, G2/M cell cycle checkpoint, and tumor angiogenesis [6–11]. *XAF1* transcription is activated in response to diverse cytokine and cytotoxic stresses, such as tumor necrosis factor (TNF), interferons (IFNs), hypoxia, genotoxic drugs, and γ-irradiation and its induction plays a critical in the apoptotic switch of various stress signaling pathways [4–7, 12, 13]. *XAF1* promoter is activated by p53 and IFN regulatory factor (IRF)-1 and elevated XAF1 stabilizes p53 and IRF-1 by preventing their ubiquitination by mouse double minute 2 (MDM2) and C-terminus of Hsc70-interacting protein (CHIP), respectively [14, 15]. XAF1 also stimulates apoptotic phosphorylation of p53 by blocking Siah2-mediated ubiquitination of homeodomain-interacting protein kinase 2 (HIPK2) while it terminates the cell-cycle arrest role of p53 by promoting zinc finger protein 313 (ZNF313)-mediated ubiquitination of p21^WAF1^ [14, 16]. In breast cancer cells, XAF1 drives an apoptotic switch of estrogen function by facilitating breast cancer-associated gene 1 (BRCA1) interaction with and ubiquitination of estrogen receptor-α (ERα) [17]. Under endoplasmic reticulum stress conditions, XAF1 destabilizes GRP78 by recruiting ZNF313 and induces an autophagy to apoptosis transition of unfolded protein response-dictated cell-fate decisions [18]. These indicate that XAF1 evokes its pro-apoptotic effect by stabilizing and/or destabilizing signaling proteins through the interplay with multiple ubiquitin E3 ligases.

Protein functions are controlled by site-specific post-translational modifications, such as phosphorylation, ubiquitylation, acetylation, and SUMOylation [19]. Ubiquitylation is sequentially proceeded by ubiquitin-activating enzymes (E1s), ubiquitin-conjugating enzymes (E2s), and ubiquitin ligases (E3s), which ubiquitylate protein substrates to mark them for proteasomal degradation. Ubiquitin E3 ligases are sub-classified into three groups: really interesting new gene (RING) and UFD2 homology (U-box) family, homologous to E6AP carboxy terminus (HECT) family, and RING-between-RING (RBR) family [20]. By dictating the fate of substrates, ubiquitin E3 ligases regulate a diverse set of proteins, thereby affecting various cellular processes, including cell proliferation and death, metabolism, homeostasis, and cellular response to external stresses. Deregulation of ubiquitin E3 ligases due to genetic and epigenetic alterations are commonly observed in human cancers and results in abnormal expression, activity, subcellular localization, and assembly of many proteins [21]. Ubiquitin E3 ligases can elicit oncogenic or tumor-suppressive activity, however their function can be diverted from tumor suppressor to oncogene or vice versa upon altered expression or post-translational modifications [22].

The transcriptional intermediary factor 1 (TIF1) family of chromatin-binding proteins is a subfamily of the large tripartite motif (TRIM) family of ubiquitin E3 ligases that is characterized by the presence of a conserved N-terminal RBCC module consisting of a RING domain, one or two B-Boxes (B1/B2) and a coiled-coil (CC) domain [23, 24]. The TIF1 family comprises TRIM24, TRIM28, TRIM33 and TRIM66 and are involved in many fundamental biological processes. All TIF1 proteins are often dysregulated or mutated in multiple human cancers and exhibit both tumor-promoting and tumor-suppressive roles depending on cell type and function [23–25]. TRIM28, also known as transcriptional intermediary factor 1β (TIF1β) and Kruppel-association box (KRAB)-associated protein 1 (KAP1), is a ubiquitously expressed protein that is involved in diverse cellular processes, including epigenetic transcription regulation, cell growth and death, DNA repair, and anti-viral defense [26]. TRIM28 functions as a transcriptional repressor by associating with KRAB domain transcription factors and can repress genomic transcription by establishing heterochromatin through macromolecular complex comprising Mi2α, SETDB1, NuRD, and HP1 and interaction with histone methyltransferases and deacetylases [26–29]. Depending on post-translational modifications, TRIM28 exerts diverse functionality. SUMOylated TRIM28 can induce gene silencing by assembling epigenetic machinery while phosphorylated and deacetylated TRIM28 is involved in DNA repair [26, 30]. As a ubiquitin E3 ligase, TRIM28 destabilizes tumor suppressor proteins, such as AMP-activated protein kinase (AMPK), p53, and LIM domain interacting (RLIM) while as a E3 SUMO-ligase, it SUMOylates IRF-7 to reduce its activity as a transcriptional activator [31–35].

Aberrant overexpression of TRIM28 is frequently observed in human cancers and associated with aggressive clinical features and poorer survival of patients in many cancers, including liver and ovarian cancer [36–39]. TRIM28 interacts with UBE2S to ubiquitinate and degrade p27, a cell cycle inhibitor, thereby stimulating growth of hepatocellular carcinoma [40]. TRIM28 promotes epithelial-to-mesenchymal transition (EMT) and metastasis of tumor cells through stabilization of Twist-related protein 1 (TWIST1) and activation of Wnt/β-catenin signaling [41–43]. It also protects tumor cells from genotoxic stress by enhancing DNA damage response, such as non-homologous end-joining repair [26, 30]. SIRT1-mediated deacetylation of TRIM28 stabilizes its interaction with 53BP1, a key DNA damage response protein, leading to increased 53BP1 concentrate formation at sites of DNA damage [30]. TRIM28 also participates in the maintenance of pluripotency of stem cells. It interacts with EZH2 and SWI/SNF chromatin remodeling complex to activate genes that are involeved in mammosphere formation, supporting its crucial role in the regulation of cancer stem cell properties [44].

Although a growing body of evidence indicates that tumor suppression function of XAF1 stems mainly from its activity to control ubiquitin E3 ligases, only a few XAF1-binding E3 ligases have been identified. In the present study, we found that XAF1 destabilizes TRIM28 to suppress tumor cell malignancy whereas TRIM28 destabilizes XAF1 to protect tumor cells from apoptotic stresses. Therefore, our study uncovers a novel mechanism underlying XAF1-mediated tumor suppression and the XAF1-TRIM28 mutual antagonism.

## Results

### XAF1 destabilizes TRIM28 through ubiquitin-mediated proteasomal degradation

**Fig. 1:**
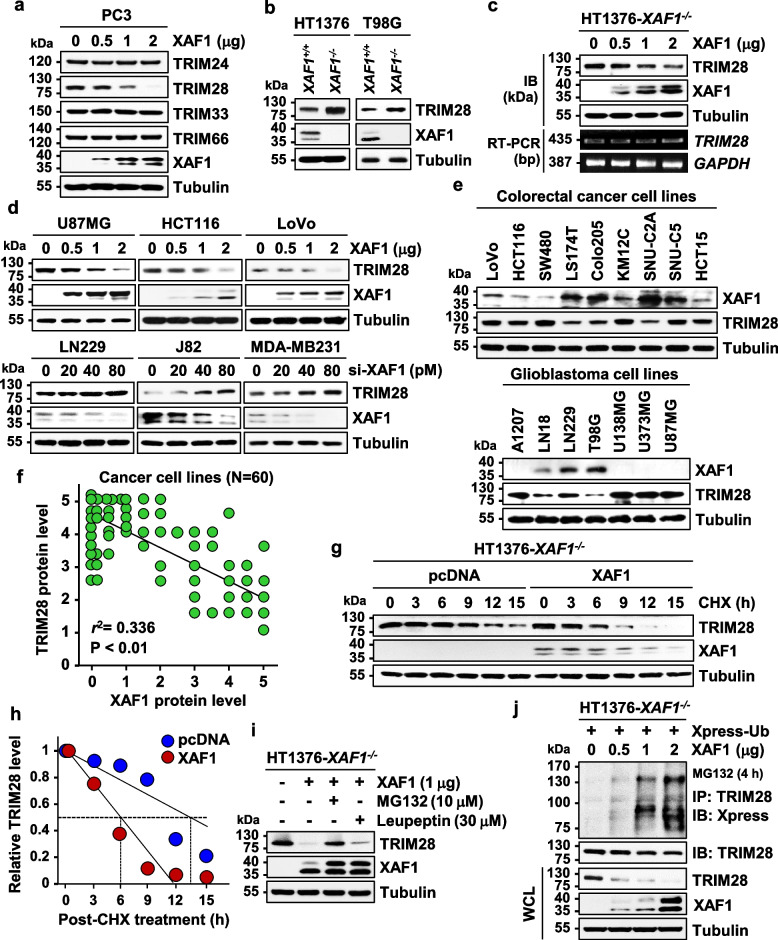
XAF1 promotes ubiquitination and proteasomal degradation of TRIM28. **a** XAF1 downregulation of TRIM28. Cells were transfected with increasing doses of XAF1 as indicated and IB assay was performed at 48 h after transfection. **b** Comparison of TRIM28 expression level in *XAF1*^+*/*+^ and *XAF1*^*−/−*^ sublines of cancer cell lines. **c** A rescue assay using HT1376-*XAF1*^*−/−*^ cells showing effect of XAF1 restoration on TRIM28 expression. RT, reverse transcription **d** Effect of XAF1 expression and depletion on TRIM28 in multiple cancer cell lines. **e** Expression status of XAF1 and TRIM28 in colorectal cancer and glioblastoma cell lines. **f** Inverse correlation of XAF1 and TRIM28 levels in 60 human cancer cell lines. Protein levels were measured by immunoblot assay in triplicates. Data represent the mean of triplicate assays. *r*^2^, Pearson’s correlation coefficient. **g**, **h** CHX chase assay showing XAF1-induced TRIM28 destabilization. Cells were exposed to CHX (20 μM) for indicated times. **i** Blockade of XAF1-induced TRIM28 destabilization by MG132. Cells were exposed to MG132 (10 μM), leupeptin (30 μM) for 6 h after 48 h of XAF1 transfection. **j** IP assay showing XAF1 stimulation of TRIM28 ubiquitination. Cells were transfected with Xpress-Ub and an increasing dose of XAF1. WCL whole cell lysate

To explore the possible interplay between XAF1 and TIF1 family proteins, we initially examined XAF1 effect on protein levels of four members (TRIM24, TRIM28, TRIM33, and TRIM66). An immunoblot (IB) assay of XAF1-nonexpressing PC3 (prostate) cells revealed that ectopic overexpression of XAF1 decreases expression of TRIM28 but not of TRIM24, TRIM33, and TRIM66 (Fig. 1a). Likewise, substantially higher levels of TRIM28 were observed in *XAF1*^*−/−*^ versus *XAF1*^+*/*+^ sublines of HT1376 (bladder) and T98G (glioblastoma) cells (Fig. 1b). As predicted, a rescue experiment using HT1376-*XAF1*^*−/−*^ cells showed that XAF1 decreases TRIM28 expression at the protein level (Fig. 1c and Fig. S1a). XAF1 effect on TRIM28 level was also observed in various types of tumor cell lines (Fig. 1d). Additionally, IB assay of 60 cancer cell lines and a database analysis of 82 human lung carcinomas (https://www.cbioportal.org/) identified a significant inverse correlation between XAF1 and TRIM28 levels (Fig. 1e, f and Fig. S1b). Given that XAF1 controls the stability of multiple proteins, we tested if XAF1 affects the protein stability of TRIM28 [13–17]. A cycloheximide (CHX) assay using HT1376-*XAF1*^*−/−*^ cells showed that XAF1 shortens the half-life of TRIM28 from approximately 13.5 h to 6 h (Fig. 1g, h). XAF1 destabilization of TRIM28 was disrupted by MG132 (proteasome inhibitor) but not affected by leupeptin (lysosome inhibitor), supporting that XAF1 promotes the proteasomal degradation of TRIM28 (Fig. 1i). As predicted, an immunoprecipitation (IP) assay using HT1376-*XAF1*^*−/−*^ cells revealed that XAF1 transfection increases TRIM28 ubiquitination in a dose-associated fashion (Fig. 1j).

### XAF1 attenuates tumor cell malignancy in a TRIM28-dependent manner

**Fig. 2:**
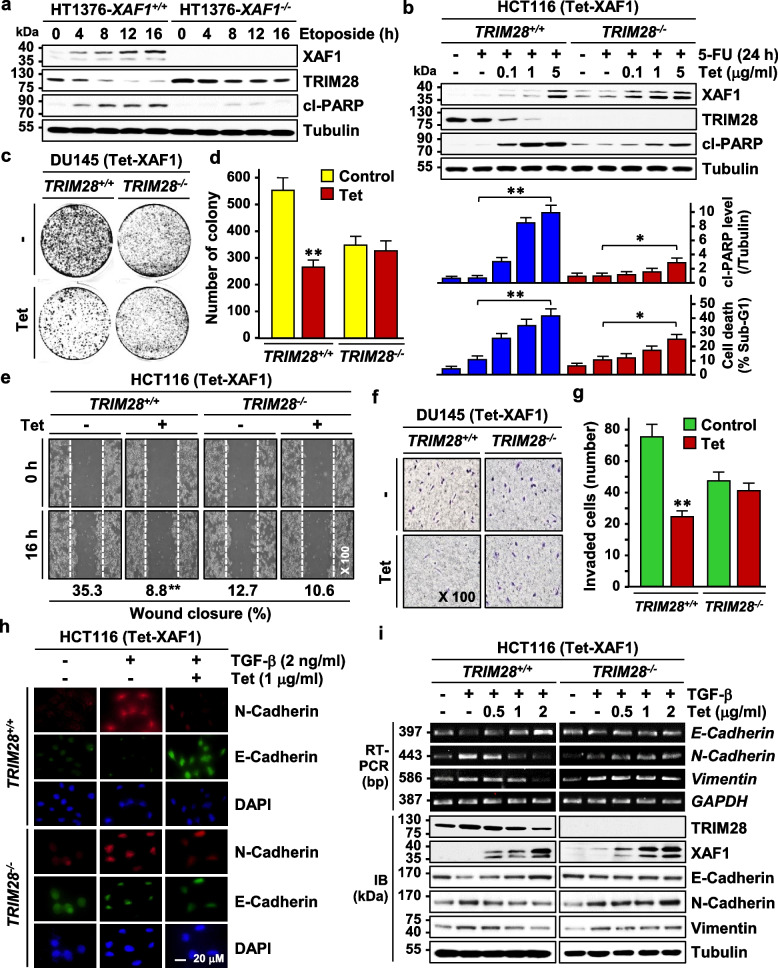
A TRIM28-dependency of XAF1 suppression of tumor cell malignancy. **a** Comparison of TRIM28 expression in *XAF1*^+*/*+^ and *XAF1*^*−/−*^ sublines of HT1376 following etoposide (25 μM) treatment. **b** Comparison of XAF1’s pro-apoptotic effect in *TRIM28*^+*/*+^ and *TRIM28*^*−/−*^ sublines of HCT116 (Tet-XAF1). XAF1 was induced by addition of tetracycline at 6 h before 5-FU (25 μM) treatment. Apoptosis was measured using flow cytometric analysis of sub-G1 fraction. Data represent the mean ± SD of triplicate assays. * *P* < 0.05; ** *P* < 0.01 (Student *t*-test). **c**, **d** Effect of TRIM28 knockout on XAF1 suppression of colony-forming ability of tumor cells. *TRIM28*^+*/*+^ and *TRIM28*^*−/−*^ sublines of DU145 (Tet-XAF1) were maintained in the presence of zeocin for 4–6 weeks. * *P* < 0.05; ** *P* < 0.01 (Student t-test). **e** Wound healing assay showing effect of *TRIM28* knockout on XAF1 suppression of tumor cell migration. Cells were exposed to tetracycline (1 μg/ml). Data represent means ± SD of triplicate assays. **f**, **g** Transwell assay showing effect of TRIM28 knockout on XAF1 suppression of tumor cell invasion. The number of invaded cells was counted by microscopic analysis. Data represent means ± SD of triplicate assays. **h**, **i** IF and RT-PCR assays of expression of EMT markers in *TRIM28*^+*/*+^ and *TRIM28*.^*−/−*^ sublines of HCT116 (Tet-XAF1). XAF1 was induced by addition of tetracycline at 6 h before TGF-β treatment (2 ng/ml, 48 h)

Next, we asked whether XAF1 tumor suppression function is linked to the TRIM28-destabilizing activity. In HT1376-*XAF1*^+*/*+^ cells undergoing etoposide-induced apoptosis, TRIM28 reduction was coupled with XAF1 induction while no detectable reduction of TRIM28 was recognized in HT1376-*XAF1*^*−/−*^ cells (Fig. 2a). Likewise, in 5-FU-exposed HCT116 (Tet-XAF1) cells and etoposide-exposed DU145 (Tet-XAF1) cells, XAF1 induction evokes much higher apoptosis-promoting effect in *TRIM28*^+*/*+^ versus *TRIM28*^*−/−*^ sublines (Fig. 2b). Based on previous reports showing that XAF1 enhances temozolomide (TMZ)-induced apoptosis via AMPK activation whereas TRIM28 destabilizes AMPK [10, 31], we tested if XAF1 activates AMPK by destabilizing TRIM28. In TMZ-exposed DU145 (Tet-XAF1) cells, XAF1 induction increased AMPK phosphorylation in *TRIM28*^+*/*+^ cells while this effect was negligible in *TRIM28*^*−/−*^ cells, supporting that XAF1 induces apoptosis in a TRIM28-dependent fashion (Fig. S2b). Moreover, colony formation, would healing, and invasion assays showed that XAF1 exerts substantially higher suppression effect on these malignant phenotypes in *TRIM28*^+*/*+^ versus *TRIM28*^*−/−*^ cells (Fig. 2c-g). Additionally, immunofluorescence (IF), RT-PCR, and IB assays of E-Cadherin, N-Cadherin, and Vimentin identified that XAF1 inhibits TGF-β induction of EMT in HCT116-*TRIM28*^+*/*+^ but not in HCT116-*TRIM28*^*−/−*^ cells (Fig. 2h, i). Together, these support that XAF1 suppresses tumor cell malignancy at least in part by targeting TRIM28.

### XAF1 attenuates the ubiquitin E3 ligase activity of TRIM28

**Fig. 3:**
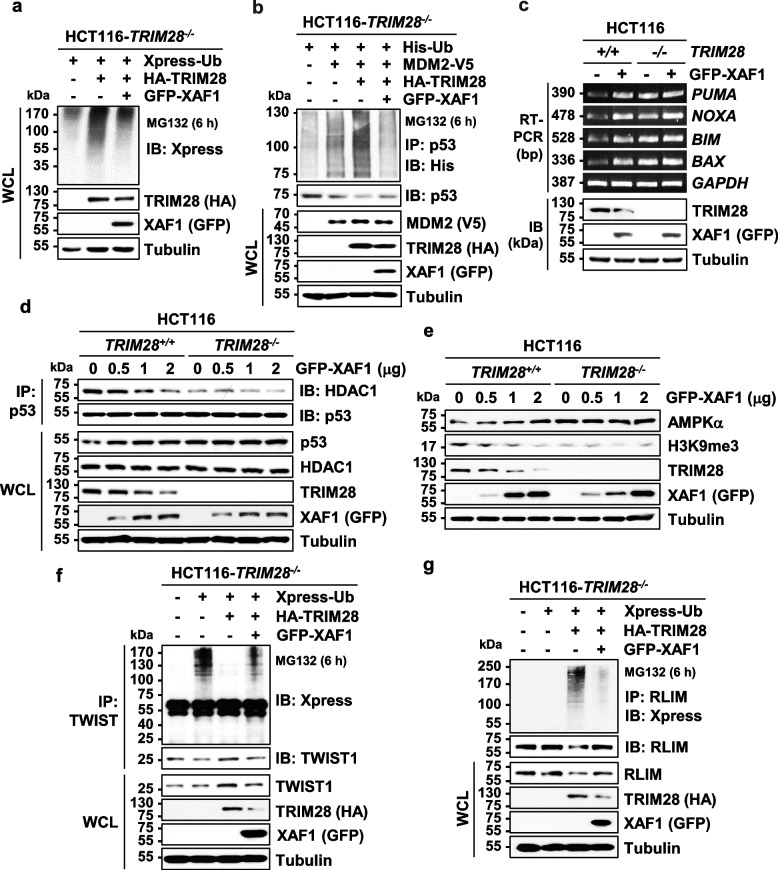
XAF1 suppresses ubiquitin E3 ligase function of TRIM28. **a** IB assay showing TRIM28-induced protein ubiquitination and its inhibition by XAF1. HCT116-*TRIM28*^*−/−*^ cells were transfected with HA-TRIM28 and GFP-XAF1 as indicated. Protein ubiquitination was examined using whole cell lysates after 48 h transfection. **b** IP assay showing TRIM28 stimulation of MDM2-mediated p53 ubiquitination and its blockade by XAF1. **c** RT-PCR assay for comparison of XAF1 effect on p53 target mRNA expression in *TRIM28*^+*/*+^ and *TRIM28*^*−/−*^ sublines of HCT116. **d** IP assay showing XAF1 effect on p53-HDAC1 interaction in HCT116-*TRIM28*^+*/*+^ and HCT116-*TRIM28*^*−/−*^ cells. **e** Comparison of XAF1 effect on expression of AMPKα and H3K9me3 in HCT116-*TRIM28*^+*/*+^ and HCT116-*TRIM28*^*−/−*^ cells. **f**,** g** IP assays showing XAF1 inhibition of TRIM28-mediated deubiquitination and RLIM ubiquitination. HCT116-*TRIM28*^*−/−*^ cells were transfected with XAF1, HA-TRIM28 and Xpress-Ub as indicated

To define if XAF1 affects TRIM28 ubiquitin E3 ligase activity that is crucial for its diverse oncogenic roles, we initially tested its effect on TRIM28-induced protein ubiquitination using HCT116-*TRIM28*^*−/−*^ cells. Protein ubiquitination was increased by TRIM28 transfection and this effect was attenuated when XAF1 was co-transfected (Fig. 3a). Given that TRIM28 stimulates p53 ubiquitination through interaction with MDM2 and represses p53 transcriptional activity via p53-HDAC1 complex formation, we tested whether XAF1 influences TRIM28-induced p53 ubiquitination and p53-HDAC1 interaction [32, 33]. As predicted, MDM2-directed p53 ubiquitination was further increased by TRIM28 and this increase was markedly abolished by XAF1 (Fig. 3b). Moreover, XAF1 suppressed p53-HDAC1 interaction and activated mRNA expression of p53 targets (*PUMA*, *NOXA*, *BIM*, and *BAX*) in *TRIM28*^+*/*+^ but not in *TRIM28*^*−/−*^ cells (Fig. 3c, d and Fig. S3a). XAF1 also up- and down-regulated AMPKα and H3K9me3, respectively in *TRIM28*^+*/*+^ but not in *TRIM28*^*−/−*^ cells (Fig. 3e and Fig. S3b). TRIM28 was reported to deubiquitinate TWIST1 and ubiquitinate RLIM to drive EMT and MDM2 elevation, respectively [34, 41]. Consistent with the reports, we observed that TWIST1 is decreased by TRIM28 transfection and RLIM is increased by TRIM28 depletion (Fig. S3c and d). IP assay using HCT116-*TRIM28*^*−/−*^ cells revealed that TRIM28-induced TWIST1 deubiquitination and RLIM ubiquitination are strongly impaired by XAF1 (Fig. 3f, g). It was also observed that XAF1 increases RLIM interaction with MDM2, which is accompanied with MDM2 reduction, in *TRIM28*^+*/*+^ but not in *TRIM28*^*−/−*^ cells, supporting that XAF1 can stabilize p53 by blocking TRIM28-mediated RLIM ubiquitination (Fig. S3e).

### XAF1 promotes TRIM28 ubiquitination via direct interaction

**Fig. 4:**
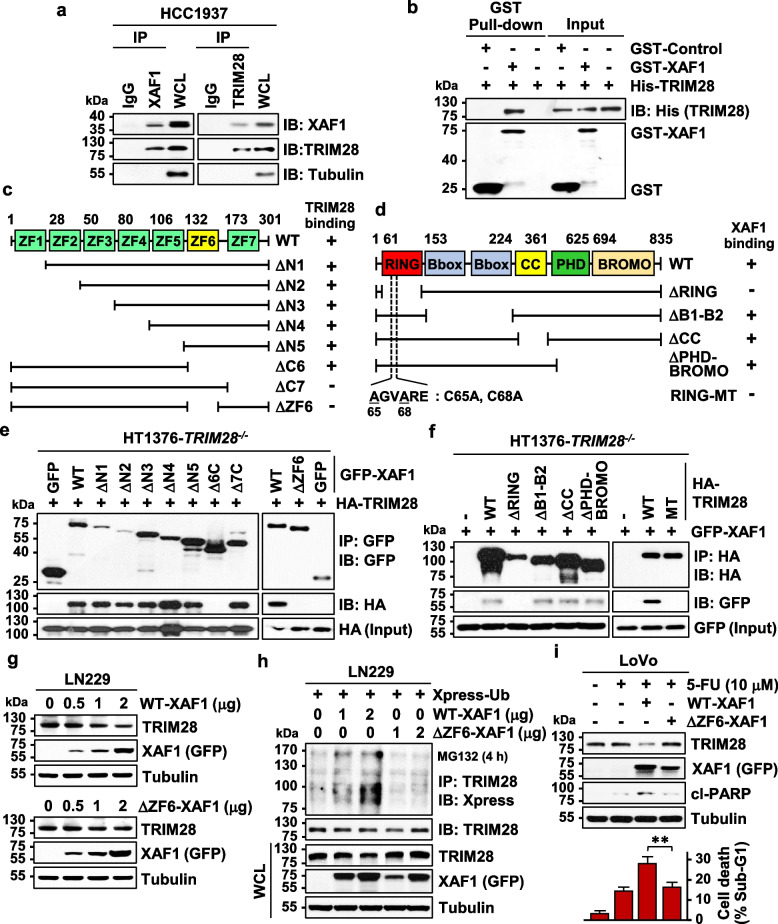
XAF1 binds directly to TRIM28. **a** IP assay showing XAF1-TRIM28 interaction in HCC1937 cells. **b** GST pull-down assay showing a direct binding of GST-XAF1 and recombinant His-TRIM28. **c** XAF1 constructs and their TRIM28-binding status. ZF zinc finger. **d** TRIM28 constructs and their XAF1-binding status. RING, really interesting new gene; CC, coiled-coil; PHD, plant homeodomain. **e** Identification of the ZF6 domain of XAF1 as a critical region responsible for TRIM28 interaction. **f** Identification of the RING domain of TRIM28 as a critical region for XAF1 interaction. **g** Comparison of TRIM28-reducing activity of WT-XAF1 and ΔZF6-XAF1. **h** Loss of TRIM28 ubiquitination-promoting activity of ΔZF6-XAF1. **i** Comparison of apoptosis-promoting activity of WT-XAF1 and ΔZF6-XAF1. Cells transfected with either WT- or ΔZF6-XAF1 were exposed to 5-FU for 24 h. Apoptosis was analyzed by IB assay of cl-PARP and flow cytometric measurement of sub-G1 fraction. Data represent the mean ± SD of triplicate assays. ** *P* < 0.01 (Student *t*-test)

To understand the molecular basis for the XAF1 destabilization of TRIM28, we tested if XAF1 interacts with TRIM28. IP assay using HCC1937 cells showed that XAF1 binds to TRIM28 but not TRIM24, TRIM33, and TRIM66 (Fig. 4a and Fig. S4a). Moreover, an in vitro binding analysis using GST-XAF1 and recombinant His-TRIM28 showed that XAF1 interacts directly with TRIM28 (Fig. 4b). IP assays using deletion mutants of XAF1 identified that the zinc finger (ZF) domain 6 of XAF1 and the RING domain of TRIM28 are required for the interaction (Fig. 4c-f and Fig. S4b). As predicted, a ZF6 deletion mutant (ΔZF6-XAF1) exhibited no activity to decrease and ubiquitinate TRIM28 (Fig. 4g, h and Fig. S4d). Moreover, a flow cytometric assay of Annexin expression revealed that ΔZF6-XAF1 exerts significantly lower apoptosis-promoting effect compared to WT-XAF1 in LoVo (colon) cells treated with 5-FU and etoposide (Fig. 4i and Fig. S4e, f).

### XAF1 stimulates ZNF313-mediated TRIM28 ubiquitination

**Fig. 5:**
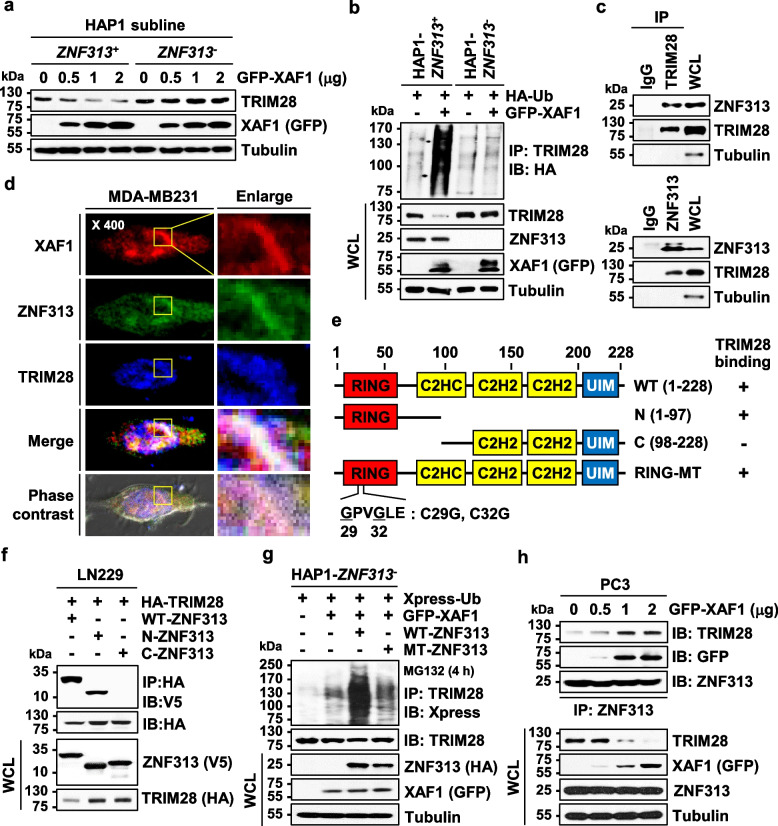
XAF1 stimulates ZNF313-mediated TRIM28 ubiquitination. **a** ZNF313 dependency of XAF1 function in TRIM28 downregulation. *ZNF313*^+^ and *ZNF313*^*−*^ sublines of the HAP1 haploid cell line were transfected with an increasing dose of XAF1. **b** Comparison of XAF1 effect on TRIM28 ubiquitination in HAP1-*ZNF313*^+^ and HAP1-*ZNF313*^*−*^ sublines. **c** ZNF313-TRIM28 interaction in HT1376 cells. **d** Immunofluorescence microscopic analysis of cellular localization of XAF1, ZNF313 and TRIM28 using anti-XAF1, anti-ZNF313, or anti-TRIM28 antibodies. **e** ZNF313 constructs and their TRIM28-binding status. UIM, ubiquitin-interacting motif. **f** Identification of the N-terminus of ZNF313 as a critical region for TRIM28 interaction. **g** Comparison of TRIM28 ubiquitination activity of WT-ZNF313 and RING-MT-ZNF313. **h** XAF1 stimulation of ZNF313-TRIM28 interaction. IP assay was carried out with an equal amount of ZNF313 input

To define E3 ubiquitin ligase(s) involved in XAF1-mediated TRIM28 ubiquitination, we examined effect of ZNF313, Siah2, CHIP, and ZNF200, which are known or predicted to interact with XAF1 [14, 16]. Among these, ZNF313 was identified to decrease TRIM28 (Fig. S5a). Moreover, XAF1-induced reduction and ubiquitination of TRIM28 were detected in *ZNF313*^+^ but not in *ZNF313*^*−*^ subline of HAP1 haploid cells (Fig. 5a, b). Likewise, XAF1-induced TRIM28 reduction was not observed in shZNF313 subline of PC3 cells (Fig. Fig. S5b). In MDA-MB231 and LN229 cells, TRIM28 was increased and decreased by ZNF313 depletion and expression, respectively (Fig. S5c). IP and IF assays showed that ZNF313 interacts and co-localizes with TRIM28 (Fig. 5c, d and Fig. S5d, e). IP assay using deletion mutants showed that ZNF313 interacts with TRIM28 via its N-terminal region including the RING domain (Fig. 5e, f). As predicted, a RING mutant (RING-MT; C29G and C32G) of ZNF313 failed to increase TRIM28 ubiquitination (Fig. 5g). XAF1 was shown to reinforce ZNF313-TRIM28 interaction, indicating that XAF1 facilitates the assembly of the ZNF313-mediated destruction complex (Fig. 5h). Consistent with our report that XAF1 binds to ZNF313 via its ZF7 domain [16], a mutant lacking the ZF7 domain (ΔZF7-XAF1) showed no activity to enhance ZNF313-TRIM28 interaction and TRIM28 ubiquitination although it appeared to retain the TRIM28-binding activity (Fig. S5f, g). In addition, it was shown that interaction between XAF1 and TRIM28 is not affected by ZNF313 depletion or knockout (Fig. S5h and i).

### TRIM28 destabilizes XAF1 by ubiquitination

**Fig. 6:**
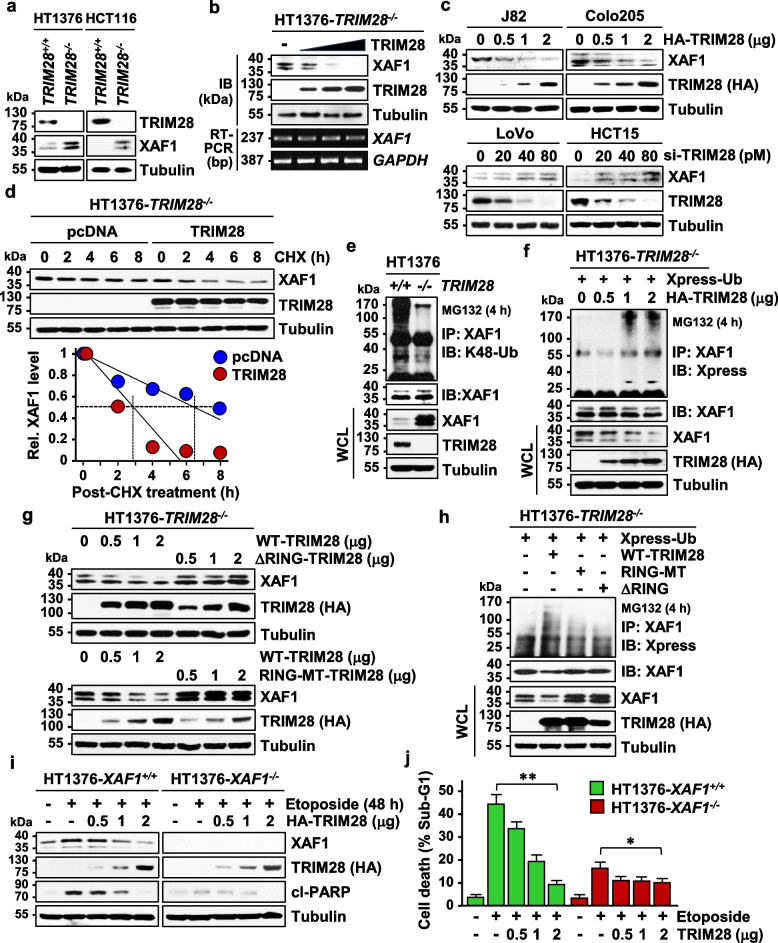
TRIM28 destabilizes XAF1 by ubiquitination. **a** Comparison of XAF1 protein level in *TRIM28*^+*/*+^ and *TRIM28*^*−/−*^ sublines of HT1376 and HCT116. **b** A rescue assay showing effect of TRIM28 restoration on XAF1 level in HT1376-*TRIM28*^*−/−*^ cells. **c** Effect of TRIM28 expression and depletion on XAF1 in human cancer cell lines. **d** CHX chase experiment showing TRIM28 destabilization of XAF1. **e** Comparison of K48-linked polyubiquitination status of XAF1 in HT1376-*TRIM28*^+*/*+^ and HT1376-*TRIM28*^*−/−*^ sublines. **f** IP assay showing TRIM28-mediated XAF1 ubiquitination. **g** Loss of XAF1-reducing activity of ΔRING-TRIM28 and RING-MT-TRIM28. IB assay was carried out at 24 h after transfection. **h** Loss of XAF1 ubiquitination activity of ΔRING-TRIM28 and RING-MT-TRIM28. **i**,** j** Effect of *XAF1* knockout on TRIM28 blockade of etoposide-induced apoptosis. Comparison of TRIM28 effect on etoposide-induced apoptosis in *XAF1*^+*/*+^ and *XAF1*.^*−/−*^ sublines of HT1376. Cells were transfected with an increasing dose of HA-TRIM28 and treated with etoposide (25 μM, 48 h). Data represent the mean ± SD of triplicate assays. * *P* < 0.05; ** *P* < 0.01 (Student t-test)

Next, we evaluated if XAF1 is affected by TRIM28 or other TIF1 family proteins. IB assay showed that XAF1 is upregulated by depletion of TRIM28 but not by TRIM24, TRIM33, and TRIM66 (Fig. S6a). XAF1 level was much higher in *TRIM28*^*−/−*^ versus *TRIM28*^+*/*+^ sublines of HT1376 and HCT116 cells and decreased in *TRIM28*^*−/−*^ cells if TRIM28 expression was restored (Fig. 6a, b). TRIM28 regulation of XAF1 was verified in multiple cancer cell lines (Fig. 6c). In response to epidermal growth factor (EGF) that induces TRIM28, XAF1 showed a more drastic reduction in *TRIM28*^+*/*+^ versus *TRIM28*^*−/−*^ subline of HT1376 (Fig. S6b). A CHX chase experiment showed that TRIM28 shortens XAF1 half-life from about 6.4 h to 3.8 h (Fig. 6d). TRIM28-induced XAF1 reduction was blocked by either MG132 or ubiquitin-activating enzyme (E1) inhibitor PYR-41, but not influenced by leupeptin, supporting that it occurs through the proteasome pathway (Fig. S6c). Moreover, K48-linked polyubiquitination level of XAF1 was substantially higher in *TRIM28*^+*/*+^ cells compared to *TRIM28*^+*/*+^ cells and elevated in *TRIM28*^*−/−*^ cells after transfection of TRIM28 (Fig. 6e, f). As predicted from Fig. 4c-f, a RING deletion mutant (ΔRING) or a RING sequence mutant (RING-MT; C-to-A replacement at codons 65 and 68) showed no activity to decrease and ubiquitinate XAF1 (Fig. 6g, h). Moreover, TRIM28 exerted more protective effect against etoposide-triggered apoptosis in *XAF1*^+*/*+^ versus *XAF1*^*−/−*^ subline of HT1376, supporting the XAF1-dependency of its anti-apoptotic function (Fig. 6i, j). Together, these indicate that TRIM28 is a XAF1-targeting ubiquitin E3 ligase, which destabilizes XAF1 in both physiological and stressful conditions.

### XAF1 promotes tumor regression in a highly TRIM28-dependent manner

**Fig. 7:**
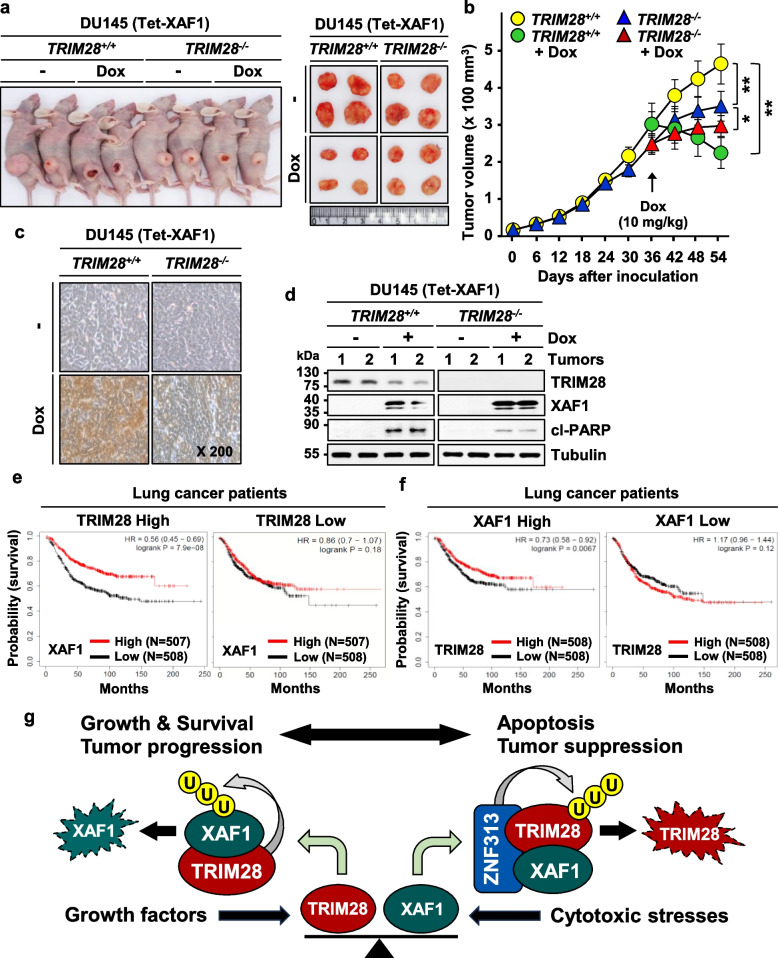
XAF1 induces tumor regression in a TRIM28-dependent manner. **a**, **b** Representative photographs of xenograft tumors derived from *TRIM28*^+*/*+^ and *TRIM28*^*−/−*^ sublines of DU145 (Tet-XAF1) cells on day 54 after inoculation. The tumors were exposed to doxycycline (Dox, 10 mg/kg) on day 36. Data represent the mean ± SD (n = 5 per group; * *P* < 0.05; ** *P* < 0.01). **c**,** d** TUNEL and IB assays showing a much higher induction of apoptosis by XAF1 in *TRIM28*^+*/*+^ versus *TRIM28*^*−/−*^ tumors. Apoptotic nuclei are stained dark brown. **e**, **f** A targeted prognostic analysis of lung cancer patients using the TCGA database of XAF1 and TRIM28 expression. **g** Schematic representation of the XAF1-TRIM28 antagonistic interplay. XAF1 is induced by cytotoxic stresses and destabilizes TRIM28 to suppress tumor cell malignancy whereas TRIM28 is activated by growth factors and destabilizes XAF1 to promote tumor progression

To elucidate a role for the XAF1-TRIM28 axis in tumor growth, mouse tumor xenograft assays were performed using *TRIM28*^+*/*+^ and *TRIM28*^*−/−*^ sublines of DU145 (Tet-XAF1). Both *TRIM28*^+*/*+^ and *TRIM28*^*−/−*^ tumors were exposed to doxycycline (Dox) for XAF1 induction at day 36 after inoculation and tumor growth was compared. As predicted, a higher growth rate was identified in *TRIM28*^+*/*+^ versus *TRIM28*^*−/−*^ tumors (Fig. 7a, b). Following Dox injection, *TRIM28*^+*/*+^ tumors displayed a significantly higher regression rate compared to *TRIM28*^*−/−*^ tumors (57% versus 13.7%) (Fig. 7a, b). TUNEL and cl-PARP immunoblot assays of tumor tissues showed that apoptosis-inducing effect of XAF1 is substantially lower in *TRIM28*^*−/−*^ tumors compared to *TRIM28*^+*/*+^ tumors, supporting the TRIM28-dependency of its growth inhibition function (Fig. 7c, d). Finally, a targeted prognostic analysis of lung cancer patients using the TCGA database (http://kmplot.com/analysis/) identified that XAF1 expression is more strongly associated with overall survival in TRIM28-high compared to TRIM28-low patient groups and that TRIM28 expression is associated with overall survival in XAF1-high patient group, supporting that the XAF1-TRIM28 interplay plays a role in tumor progression process (Fig. 7e, f).

Collectively, we found that XAF1 destabilizes TRIM28 and thereby antagonizes its oncogenic activity to suppress tumor cell malignancy while TRIM28 destabilizes XAF1 to protect tumor cells from apoptotic stresses (Fig. 7g). Our study also uncovers that XAF1-TRIM28 mutual antagonism represents a novel mechanism that dictates cell response to various stimuli, suggesting that its alteration might contribute to tumorigenesis.

## Discussion

Despite accumulating evidence indicating that XAF1-mediated tumor suppression stems mainly from its property to interact with multiple ubiquitin E3 ligases, our understanding for XAF1-interacting E3 ligases is highly limited. We thus attempted to identify novel XAF1-interacting E3 ligases that play a key role in tumorigenic process. In this study, we report TRIM28 ubiquitin E3 ligase as a novel destabilization target of XAF1. By recruiting TRIM28 to another E3 ligase ZNF313, XAF1 promotes ubiquitination and proteasomal degradation of TRIM28, thereby antagonizing its tumor-promoting activity. Therefore, our study establishes XAF1 as an intrinsic antagonist of TRIM28, illuminating a novel mechanism underlying its tumor suppression function.

Aberrant overexpression of TRIM28 is frequently observed in multiple human cancers and associated with poorer survival of patients, indicating that it plays a critical role in malignant tumor progression [36–39]. Nevertheless, the molecular mechanism underlying tumor-specific elevation of TRIM28 remains largely uncharacterized. In the current study, we observed that TRIM28 elevation is associated with abnormal reduction of XAF1 in cancer cell lines and tumor tissues. Our study also provides evidence that TRIM28 elevation due to XAF1 inactivation protects tumor cells from apoptotic stresses. Considering that aberrant overexpression of TRIM28 contribute to various aspects of tumor malignancy, including enhanced growth, invasion, metastasis, and therapeutic resistance, our finding raises the possibility that increased stability and activity of TRIM28 in tumor cells harboring epigenetic silencing of *XAF1* transcription might be a critical event to promote tumor progression.

XAF1 is expressed ubiquitously in normal tissues but markedly downregulated or undetectable in a substantial faction of cancer cell lines and primary tumors due to aberrant promoter hypermethylation [2–5]. However, in certain tumor cells with no aberrant promoter hypermethylation, XAF1 protein is hardly detectable, indicating the post-transcriptional inactivation of XAF1. Moreover, XAF1 protein is degraded rapidly after synthesis and thus maintained at very low level under physiological conditions [14–18]. However, the mechanism for the maintenance of low steady-state level of XAF1 protein has not been elucidated. Interestingly, we identified TRIM28 as a XAF1-targeting ubiquitin E3 ligase. The RING domain of TRIM28 binds XAF1 to direct K48-linked polyubiquitination under both physiological and stressful conditions. Therefore, it is highly plausible that TRIM28 might be responsible for low steady-state level of XAF1 under physiological conditions and that its tumor-specific overexpression might be an alternative mechanism leading to XAF1 inactivation in cancer cells, particularly with no promoter hypermethylation.

It is well-documented that as a ubiquitin E3 ligase, TRIM28 contributes to tumorigenesis by destabilizing tumor suppressor proteins and stabilizing oncogenic proteins [29]. TRIM28 destabilizes AMPK, p53, and RLIM to protect tumor cells from apoptotic stresses while it stabilizes TWIST1 and activates Wnt/β-catenin signaling to promote cell cycle progression, EMT, and metastasis [31–34, 40–43]. Interestingly, we found that XAF1 is a novel substrate of TRIM28 and that EGF-induced TRIM28 protects tumor cells from etoposide-induced apoptosis more significantly in *XAF1*^+*/*+^ versus *XAF1*^*−/−*^ cells, indicating that TRIM28 exerts anti-apoptotic effect in a highly XAF1-dependent fashion. Therefore, our study adds XAF1 to TRIM28’s targets of tumor suppression function and identifies a novel mechanism for TRIM28-driven tumor progression. Considering that XAF1 is involved in various cellular processes, including proliferation, autophagy, and angiogenesis as well as apoptosis, TRIM28 may drive diverse malignant phenotypes by targeting XAF1 [8–11]. Collectively, our study identifies the XAF1-TRIM28 mutual antagonism and its alteration in tumorigenesis. In this context, it is noticeable that XAF1 level correlates more strongly with overall survival in TRIM28-high versus TRIM28-low patient groups, supporting the interplay of XAF1 and TRIM28. Although clinical data we analyzed in this study are very limited, it was also recognized that TRIM28-high patients show better overall survival in XAF1-high group. Given that XAF1 is a stress-inducible growth inhibitor, it is likely that stress-induced high XAF1 could activate its tumor suppression signaling through TRIM28 destruction more efficiently in TRIM28-high versus TRIM28-low tumors. Further studies will be required to understand the clinical implication of XAF1-TRIM28 interplay alteration.

Our previous studies demonstrated that the ZF7, ZNF6, and ZF4 domains of XAF1 bind to the RING domains of ZNF313, Siah2, and BRCA1, respectively, indicating that XAF1 has a property to interact with RING family ubiquitin E3 ligases through different ZF domains [14–18]. Through the interaction with ZNF313 and BRCA1, XAF1 reinforces their interaction with and ubiquitination of GRP78 and ERα, respectively [17, 18]. In contrast, through the interaction with Siah2 and CHIP, XAF1 blocks their interaction with and ubiquitination of HIPK2 and IRF-1, respectively [14, 15]. These findings show that XAF1 binds to the RING domains of ubiquitin E3 ligases to control their accessibility to target proteins. Consistently, we identified that XAF1 interacts with the RING domain of TRIM28 through the ZF6 domain. Intriguingly, however, XAF1 does not control the target accessibility of TRIM28 but destabilizes it directly by recruiting ZNF313, indicating that XAF1 acts as a bridge linking two ubiquitin E3 ligases to destruct an oncogenic ubiquitin E3 ligase. Considering that XAF1 has multiple ZF domains (ZF1-ZF7), our data strongly support that XAF1 may act as a bridge or adaptor molecule to control the stability of multiple tumor-associated ubiquitin E3 ligases.

The TIF1 family of ubiquitin E3 ligases comprising TRIM24, TRIM28, TRIM33 and TRIM66 is defined by the presence of a conserved RBCC module, one or two B-Boxes and a coiled-coil domain [23, 24]. Except for TRIM66, all the other members have one RING domain within a N-terminal RBCC module. Despite their structural similarity and frequent dysregulation in multiple human cancers, only TRIM28 shows the property to interact with XAF1, suggesting that XAF1 may recognize unique amino acid sequences in the RING domain of TRIM28 [23–25]. The overall sequence identity in the RBCC module consisting of RING domain between TRIM28 and the other members (TRIM24, TRIM33, and TRIM66) is 40%, 43%, and 29%, respectively [26]. Moreover, only 35 of 63 (56%) RING sequences of TRIM28 are shared with those of TRIM24 and TRIM33, supporting that sequence difference in RING domain might be a primary cause for the TRIM28-specific interaction with XAF1. Further molecular studies will be required to define whether XAF1 can bind and regulate other families of ubiquitin E3 ligases carrying HECT, U-box, and PHD-finger.

IRF-1 plays a crucial role in the regulation of immune response, viral infection, and inflammation [45]. IRF-1 also functions as a pro-apoptotic tumor suppressor through the transcriptional regulation of genes involved in cell proliferation, apoptosis, and angiogenesis. We reported previously that *XAF1* is induced as a direct target of IRF-1 and elevated XAF1 interacts with IRF-1 to prevent CHIP-mediated ubiquitination, indicating that XAF1 acts as a transcriptional coactivator of IRF-1 by forming a positive feedback loop [15]. We also showed that XAF1 forms a complex with IRF-1 in the nucleus to reinforce IRF-1-drived gene transcription. Interestingly, a recent study identified that XAF1 acts as an epigenetic regulator to promote expression of genes involved in antiviral defense by liberating repressed chromatin [46]. Upon RNA virus infection, MAVS recruits XAF1 and TBK1, and XAF1 phosphorylated by TBK1 is translocated to the nucleus. XAF1 then interacts with TRIM28 with the guidance of IRF-1 and inhibits SUMOylation of TRIM28 to enhance induction of antiviral genes. Therefore, XAF1 modifies chromatin structure to enhance antiviral immunity by targeting TRIM28 during infection. In this context, our finding of XAF1 destabilization of TRIM28 further supports that XAF1 acts as a key intrinsic antagonist against TRIM28 functions in various cellular contexts, including genotoxic and viral stress conditions.

Collectively, this study identifies XAF1 as a novel antagonist of TRIM28 and the presence of mutual antagonism between XAF1 and TRIM28, raising the possibility that the restoration of balanced XAF1-TRIM28 interplay could be an attractive avenue for the therapeutic intervention of tumor progression. A better understanding of the mechanisms underlying the role for XAF1 in the selective regulation of ubiquitin pathways is expected to provide novel opportunities for therapeutic intervention based on the identification of ubiquitin E3 ligases as desirable drug targets.

## Materials and methods

### Cancer cell lines reagents

Human cell lines were purchased from American Type Culture Collection (Rockville, MD, USA) and Korea Cell Line Bank (Seoul, Korea). All the cell lines were authenticated by short tandem repeat profiling at the Korea Cell Line Bank before use and tested regularly for *Mycoplasma* using PCR Mycoplasma Detection Kit (MycoStrip™, InvivoGen, CA, USA). Cells with a tetracycline-inducible XAF1 system was generated as previously described [14–18]. The *XAF1* knockout cells were generated using the CRISPR/Cas9 system. The cells were transfected with spCas9 protein (Innolifetech) and sgRNA (5’-AGCCUCAUGGAGGGUGAAGU-3’) targeting the exon 1 region of the gene using NEON™ (Thermo Fisher Scientific) electroporation system. The *TRIM28* knockout cells were generated using sgRNA (5’-GAAGCACUGUUGCUUGCACA-3’) targeting the exon 2 region of the *TRIM28* gene. Genotype was validated by Guide-it™ Genotype Confirmation Kit (Takara) according to the manufacturer’s protocol. PYR-41, cycloheximide (CHX), leupeptin, etoposide and 5-FU were purchased from Sigma-Aldrich (Saint Louis, MO, USA). MG132 (HY-13259) and recombinant human TGF- β1 were obtained from AG Scientific Inc (CA, USA) and R&D Systems (MN, USA), respectively.

### Expression plasmids and siRNAs

Plasmids encoding *XAF1*, *TRIM28,* or *ZNF313* were constructed as previously described [4, 5, 16]. siRNAs against *XAF1* (5’-ATGTTGTCCAGACTCAGAG-3’), *TRIM28* (5’- CCATGATGCCCAGAAGGTGA-3’), *TRIM24* (5’-GCUGGACUCUCUAAACAAUTT-3’), *TRIM33* (5’-AACTGGAAAGTAATCAGTCGC-3’), *TRIM66* (5’-GCAGAGCCTTCGAGATAAA-3’), and *ZNF313* (5’-GCUGCCGUAAGAAUUUCUU-3’) were synthesized by Bioneer Inc (Daejeon, Korea). Control siRNAs were obtained from Dharmacon Research (CO, USA) and Ambion (AM4635). Transfection was carried out using Neon® Transfection System (Thermo Fisher Scientific), Lipofectamine 2000 (Invitrogen), or E-fection Plus Reagent (Lugen Sci).

### Semi-quantitative reverse transcription-PCR

Total cellular RNA was harvested with easy-BLUE (iNtRONBiotechnology, Seongnam, Korea) and reverse transcription was carried out using M-MLV reverse transcriptase (Thermo Fisher Scientific). PCR was performed with TaKaRa Taq™ DNA polymerase (R001A) using primers for *XAF1, TRIM28, E-Cadherin, N-Cadherin, Vimentin, PUMA, NOXA, BIM, BAX* and *GAPDH*. Primer sequences are available upon request.

### Apoptosis analysis

Flow cytometric analysis of apoptosis was carried out using FACScan flow cytometer. For the measurement of sub-G1 fraction, cells were incubated with PBS containing 100 mg/ml RNase and 50 mg/ml propidium iodide. Annexin V analysis was performed using Annexin V-FITC Detection kit (APOAF) (Sigma-Aldrich). Annexin V-positive and PI-positive cell percentages were determined by BD Accuri C6 (BD Biosciences). TUNEL assay for apoptotic cells in xenograft tumor tissues was carried out using a DeadEnd™ Colorimetric TUNEL System Kit (Promega #G7360).

### Immunoblot and immunoprecipitation assay

Antibodies specific for XAF1 (SC-19194, SC-374378, SC-398012 and CST #13,850), TRIM28 (ab22553 and CST #4123), TRIM24 (ab70560), TRIM33 (CST #8972), TRIM66 (SC-515177), cleaved PARP (CST #9541), HA (SC-7392 and SC-805), GFP (SC-8334 and SC-9996), V5 (SC-58052 and SC-83849), Myc (SC-40), His (SC-803), GST (SC-33613), Ub (CST #8081), Xpress (Invitrogen #R910-25), RLIM (SC-101117), TWIST (SC-81417), p53 (SC-126), MDM2 (SC-813), AMPKα (CST #2532S), HDAC1 (CST #2062S) and β-tubulin (T8328) were obtained from Santa Cruz Biotechnology, Cell Signaling Technology, Merck Millipore, Abcam and Sigma-Aldrich. Detection of antibody binding was carried out using enhanced chemiluminescence (Amersham Biosciences) and a secondary antibody conjugated to horseradish peroxidase. Quantitation of XAF1 and TRIM28 levels was performed by densitometric scanning of bands on X-ray film and integration analysis using Quantity One software program (Bio-Rad). Quantitation was repeated at least 3 times for each cell line, and the mean was obtained.

### GST pull-down assay

In vitro binding assay was carried out as previously described [16, 47]. Briefly, GST-fused XAF1 proteins were purified by Glutathione Sepharose 4B (GE Healthcare), and human recombinant His-TRIM28 (H00010155-P01) was purchased from Novus biologicals. GST-XAF1 and recombinant His-TRIM28 were incubated, and immunocomplexes separated by protein-A/G Sepharose were subjected to SDS/PAGE analysis.

### Immunofluorescence

Cells fixed with 2% paraformaldehyde in PBS were permeabilized with 0.2% Triton X-100 and blocked with 3% BSA. The cells were incubated overnight with anti-GFP (SC-9996), anti-XAF1 (SC-374020, ab17204, Abcam), anti-TRIM28 (CST #4123), anti-N-Cadherin (SC-59987), anti-E-Cadherin (CST #3195) or anti-ZNF313 (SC-101116) antibody at 4 °C and stained with secondary antibodies. The fluorescence images were detected by a confocal microscope (LSM-800, Carl Zeiss MicroImaging Inc).

### Ubiquitination assay

Cells were transfected with either HA-Ub or Xpress-Ub using an E-fection reagent (Lugen Sci, Bucheon, Korea). MG132 (10 μM) was added to the cells for 4 h or 6 h before harvest. Cell lysates were prepared using a buffer containing complete protease inhibitor (Roche) and deubiquitinase inhibitor *N*-Ethylmaleimide (NEM) (E3876, Sigma-Aldrich) and incubated with anti-XAF1, anti-TRIM28, anti-HA, or anti-Xpress antibody. Ubiquitin conjugated proteins were eluted by boiling in 2X protein sample buffer and separated by SDS/PAGE.

### Mouse xenograft assays

Identical numbers (1 × 10^6^) of *TRIM28*^+*/*+^ and *TRIM28*^*−/−*^ subline cells of DU145 (Tet-XAF1) were injected subcutaneously into 5-week-old immunodeficient female nude mice (nu/nu) (Orient Bio Inc., Seongnam, Korea). Tumor growth was monitored every 6 days after inoculation. Tumor volume (V) was measured using the modified ellipsoidal formula: V = 1/2 × length × (width)^2^. At day 36, mice were exposed to doxycycline (10 mg/kg) by intratumoral injection and tumor growth was compared regularly. All animal studies were carried out with the approval (KUIACUC 2021–0044) of the Korea University Institutional Animal Care and Use Committee and the Korea Animal Protection Law.

### Statistical analysis

Gene expression, colony formation, wound healing, invasion, and apoptosis analyses were carried out in triplicates. The data were presented as mean ± SD and the Student *t*-test (GraphPad Prism5 software, CA, USA) was performed for the statistical significance. A P-value of less than 0.05 was considered significant. Pearson correlation coefficient (*r*) was used to measure the strength of the association.

## Supplementary Information

Supplementary Material 1: Fig. S1. a Quantitation of relative protein and mRNA levels of TRIM28. HT1376- XAF1 *−/−* cells were transfected with an increasing dose of XAF1 as indicated. Expression levels of TRIM28 protein and mRNA levels were determined at 48 h after transfection. Data represent the mean ± SD of triplicate assays. ** *P* < 0.01 (Student t -test). b cBioportal database analysis showing the inverse correlation of XAF1 mRNA and TRIM28 protein levels in 82 human lung carcinoma tissues. TRIM28 protein and XAF1 mRNA expression were measured by proteomics and transcriptomics analyses, respectively. r 2 , Pearson’s correlation coefficient. Fig. S2. a Comparison of XAF1 effect on etoposide-induced apoptosis in TRIM28 +*/*+ and TRIM28 *−/−* sublines of DU145 (Tet-XAF1) cells. XAF1 was induced by addition of tetracycline at 6 h before etoposide (25 μM) treatment. Apoptosis was determined by flow cytometric measurement of sub-G1 fraction and IB assay of cleaved PARP expression at 24 h after etoposide treatment. Data represent the mean ± SD of triplicate assays. Statistical analysis was conducted using Student ‘s t-test, as significance determined as ** *P* < 0.01. b Comparison of XAF1 effect on TMZ-mediated activation of AMPK phosphorylation and expression of cleaved CASP3 in TRIM28 +*/*+ and TRIM28 *−/−* sublines of DU145 (Tet-XAF1) cells. XAF1 was induced by addition of tetracycline at 6 h before TMZ (100 μM) treatment. Fig. S3. a, b Quantitation of p53, AMPKα, H3K9me3, and TRIM28 protein levels. HCT116- TRIM28 +*/*+ and HCT116- TRIM28 *−/−* cells were transfected with an increasing dose of XAF1 as indicated. After 48 h transfection, protein levels were determined. Data represent the mean ± SD of triplicate assays. ** *P* < 0.01 (Student t -test). c, d IB assays for effect of TRIM28 transfection and depletion on expression of TWIST and RLIM in TRIM28 *−/−* and TRIM28 +*/*+ sublines of HCT116. e IP assay for comparison of XAF1 effect on MDM2-RLIM interaction in TRIM28 *−/−* and TRIM28 +*/*+ sublines of HCT116. Fig. S4. a IP assay showing no interaction of XAF1 with TRIM24, TRIM33, and TRIM66 in HCC1937 cells. b IP assay showing loss of TRIM28-interacting activity of ΔZF6-XAF1. HCT116 cells transfected with either GFP-tagged WT-XAF1 or ΔZF6-XAF1. c IP assay showing loss of TRIM28 ubiquitination-promoting activity of ΔZF6-XAF1. d Quantitation of TRIM28 protein levels. LN229 cells were transfected with an increasing dose of either WT-XAF1 or ΔZF6-XAF1 as indicated. After 48 h transfection, TRIM28 levels were determined. Data represent the mean ± SD of triplicate assays. ** *P* < 0.01 (Student t -test). e, f Comparison of WT-XAF1 and ΔZF6-XAF1 effect on etoposide-induced apoptosis in LoVo cells. Cells were transfected with either WT-XAF1 or ΔZF6-XAF1 and exposed to etoposide (20 μM) for 16 h. Apoptosis was determined by flow cytometric measurement of Annexin V expression. Data represent the mean ± SD of triplicate assays. Statistical analysis was conducted using Student ‘s t-test, as significance determined as * *P* < 0.05, ** *P* < 0.01. Fig. S5. a Identification of ZNF313 as a TRIM28-targeting ubiquitin E3 ligase. HT1376 cells were transfected with 2 μg of expression vectors for CHIP, ZNF313, ZNF200, and Siah2 and its effect on TRIM28 protein expression was determined by IB assay at 24 h after transfection. b Comparison of XAF1 effect on TRIM28 expression in sh-Control and sh-ZNF313 sublines of PC3 cells. c Effect of ZNF3131 depletion and overexpression on TRIM28 expression in human cancer cells. d IP assay showing the interaction of overexpressed TRIM28 and ZNF313 in LN229 cells. Cells were transfected with 2 μg of HA-TRIM28 and ZNF313-V5 and IP was carried out at 24 h after transfection. e Immunofluorescence microscopic analysis of cellular localization of XAF1, ZNF313 and TRIM28 in HT1376 cells using anti-XAF1, anti-ZNF313, or anti-TRIM28 antibodies. f No activity of ΔZF7-XAF1 to reinforce ZNF313-TRIM28 interaction. IP was performed using an equal amount of HA-TRIM28 input. g IP assay showing loss of TRIM28 ubiquitination-promoting activity of ΔZF6-XAF1 and ΔZF7-XAF1. h, i IP assay showing no effect of ZNF313 depletion or knockout on XAF1-TRIM28 interaction. IP was performed using an equal amount of TRIM28 input. Fig. S6. a XAF1 upregulation by TRIM28 depletion. LoVo cells were transfected with 40 pM of siRNAs against four TIF1 family members (TRIM24, TRIM28, TRIM33, and TRIM66) and its effect on XAF1 expression was determined by IB assay at 24 h after transfection. b Comparison of EGF effect on XAF1 expression in TRIM28 +*/*+ and TRIM28 *−/−* sublines of HT1376 cells. Cells were exposed to increasing doses of EGF for 24 h as indicated. c Blockade of TRIM28-induced XAF1 destabilization by MG132 and PYR-41. HT1376- TRIM28 *−/−* cells were transfected with either 1 μg of pcDNA or TRIM28. After 48 h of transfection, the cells were exposed to MG132 (10 μM), leupeptin (30 μM), or PYR-41 (30 μM) for 6 h.

Supplementary Material 2.

## Data Availability

All data generated or analyzed during this study are included in this published article. Further inquiries can be directed to the corresponding author.

## Abbreviations

AMPK: AMP-activated protein kinase
BRCA1: Breast cancer-associated gene 1
CHIP: C-terminus of Hsc70-interacting protein
CHX: Cycloheximide
EMT: Epithelial-to-mesenchymal transition
IFN: Interferon
KAP1: KRAB-associated protein 1
RLIM: LIM domain interacting
MDM2: Mouse double minute 2
RING: Really interesting new gene
TIF1: Transcriptional intermediary factor 1
TNF: Tumor necrosis factor
TRIM28: Tripartite motif containing 28
TWIST: Twist-related protein
XIAP: X-linked inhibitor of apoptosis-associated factor 1
XAF1: XIAP-associated factor 1
ZNF313: Zinc finger protein 313
