# Supplementary figures and images for: XAF1 antagonizes TRIM28 activity through the assembly of a ZNF313-mediated destruction complex to suppress tumor malignancy
Source: Mol Biomed. 2024 Nov 13;5:58. doi: 10.1186/s43556-024-00224-9 (PMC11557793; doi:10.1186/s43556-024-00224-9)

a

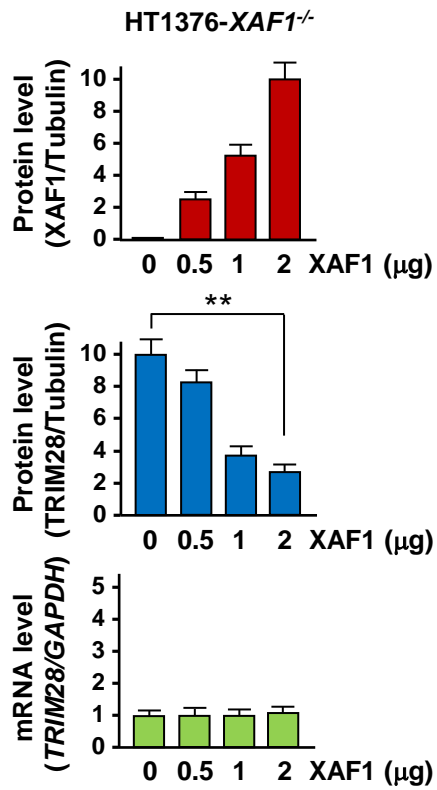

b

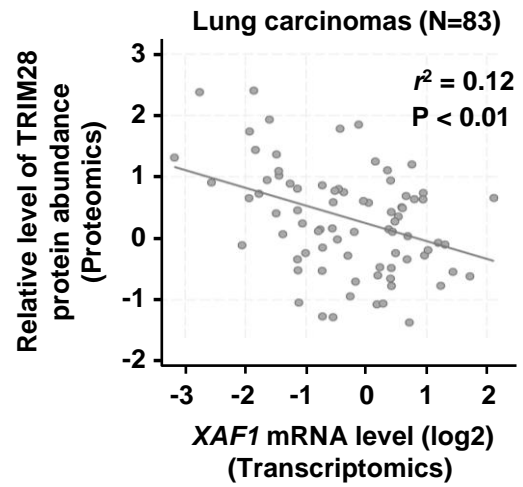

**a**

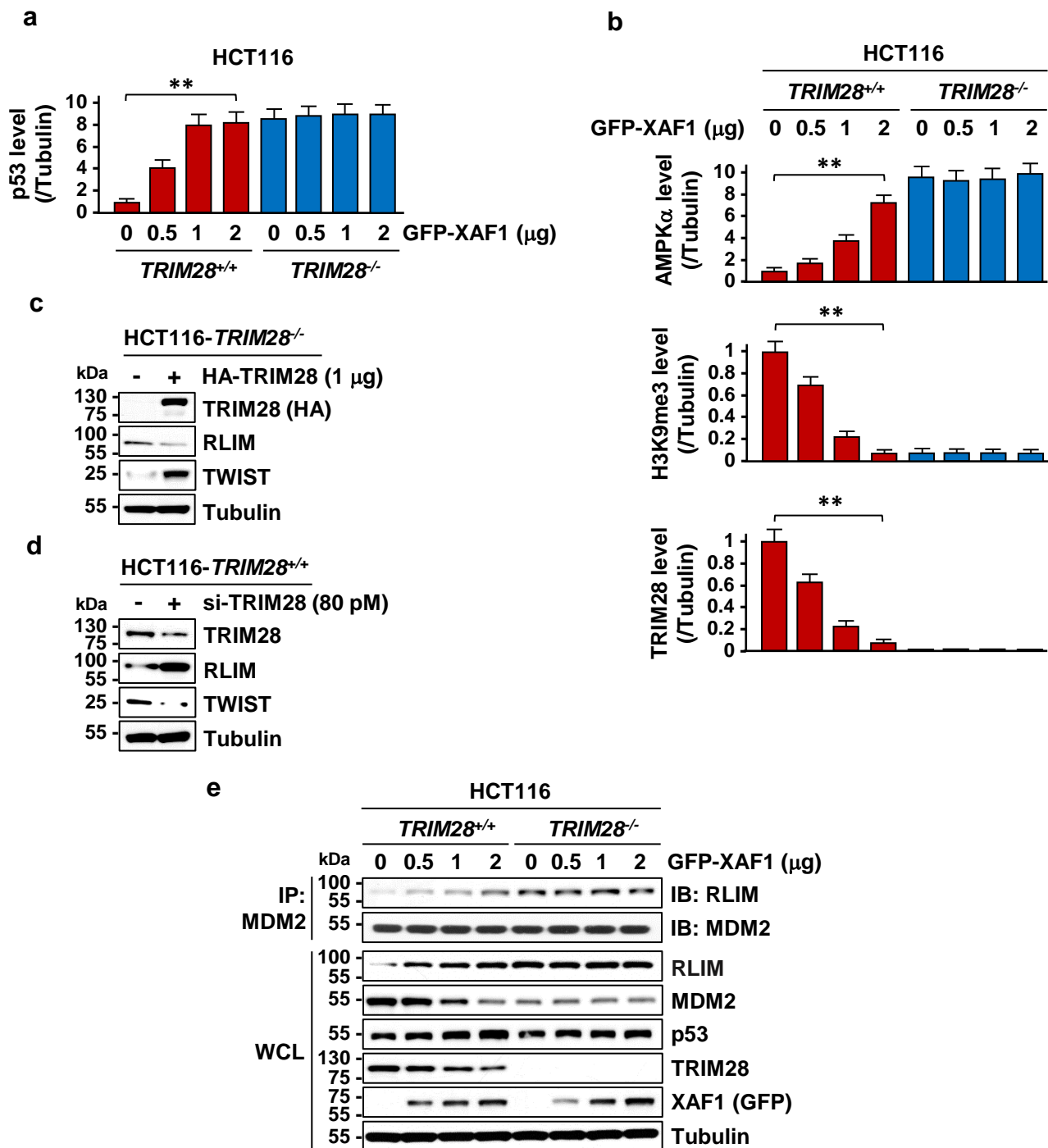

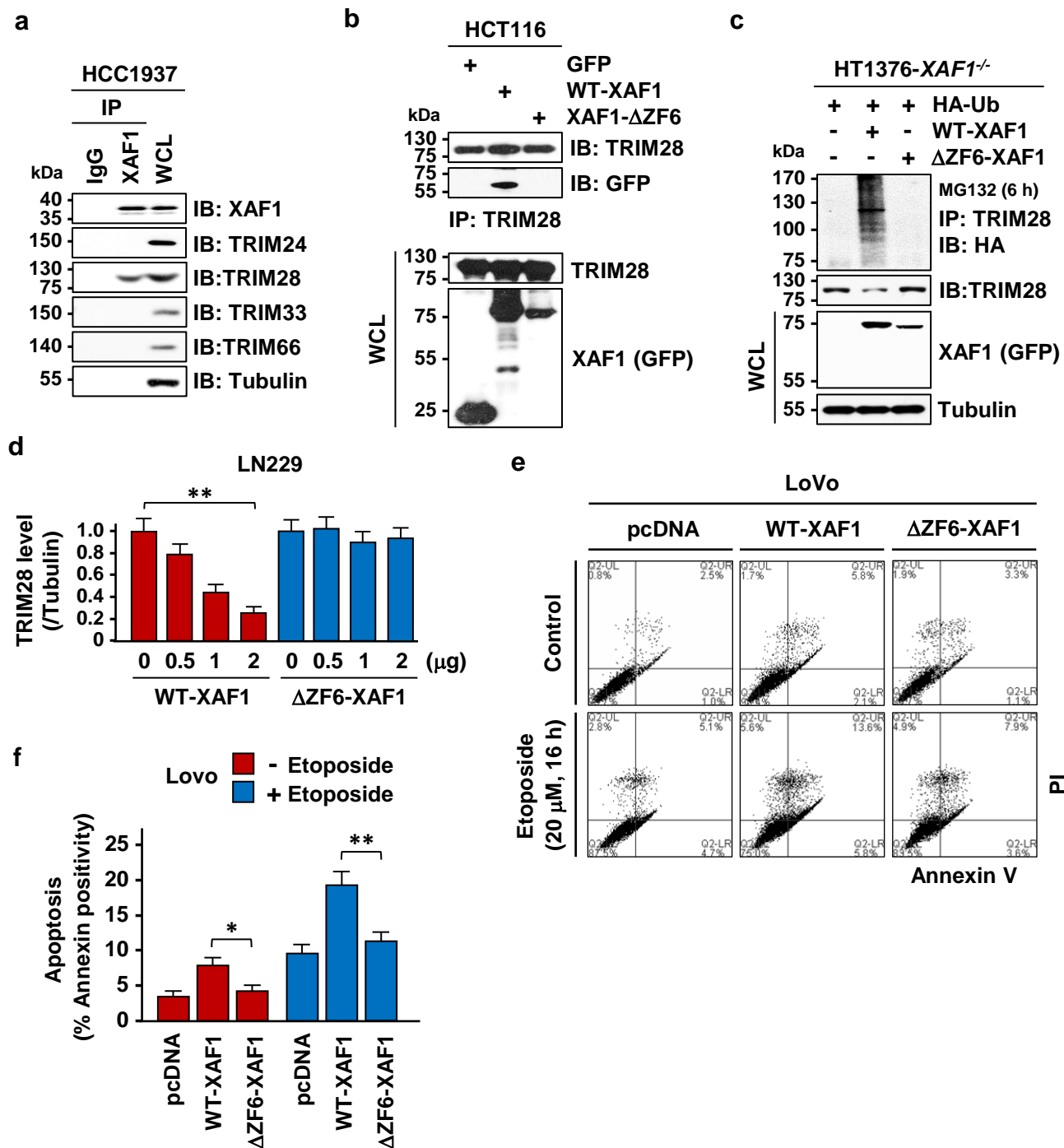

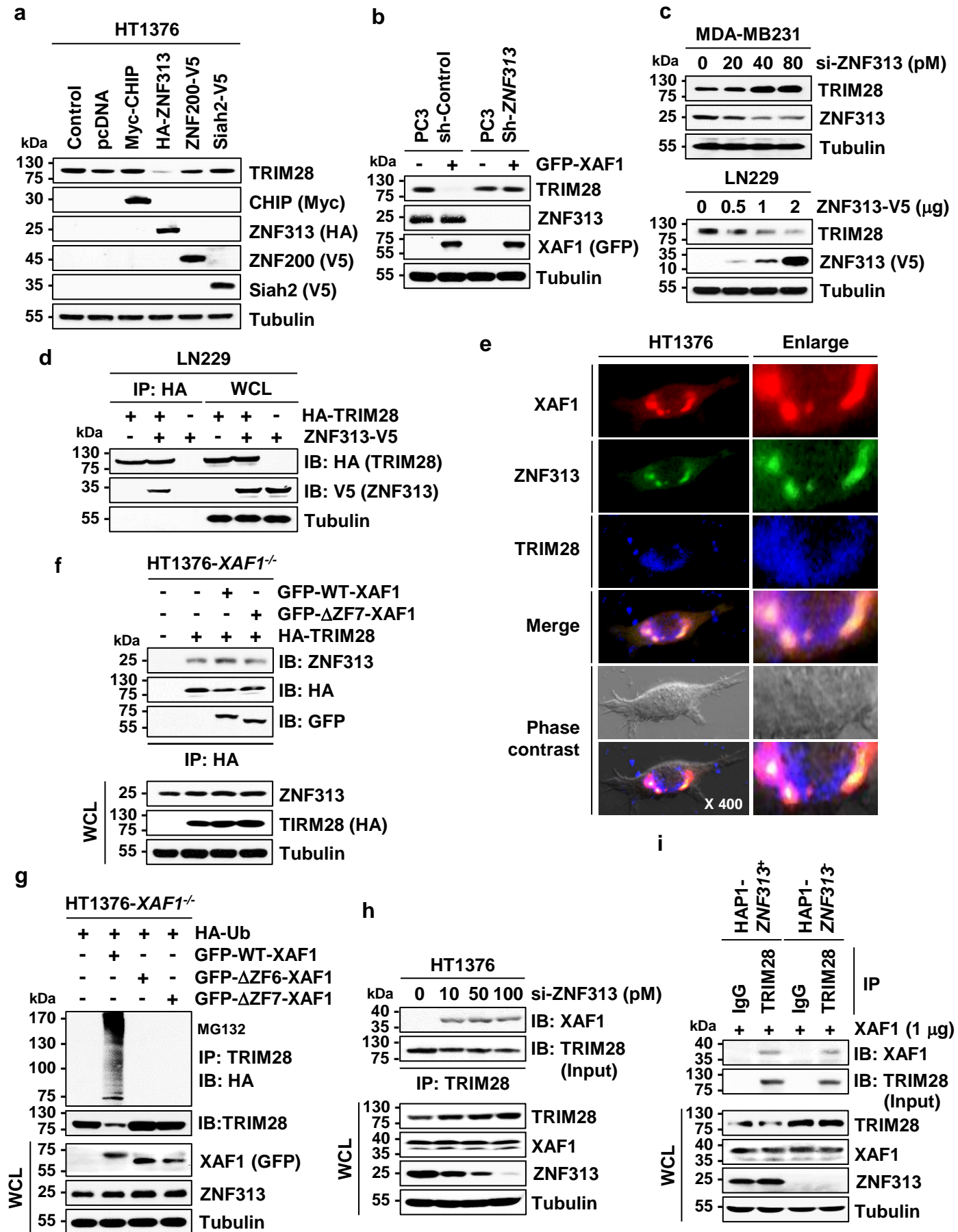

**a**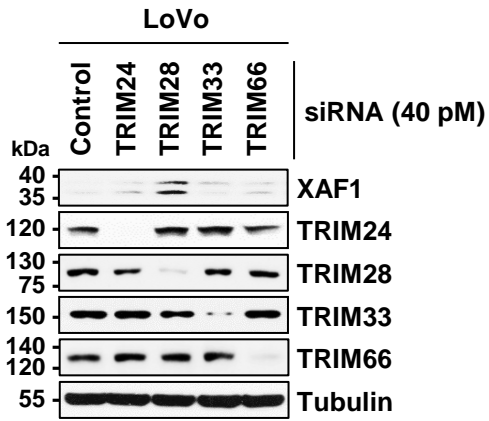**b**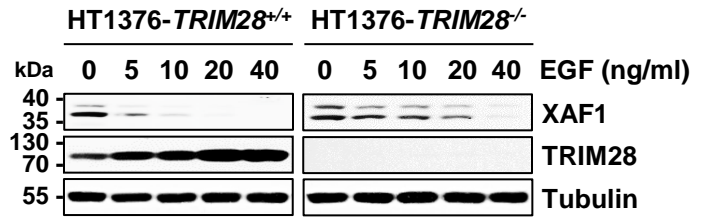**c**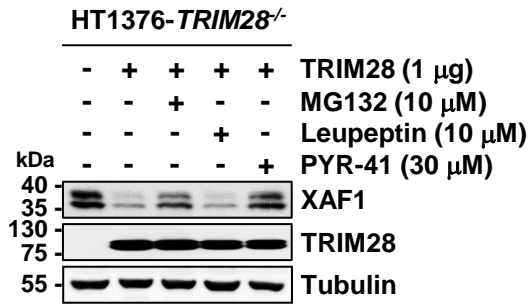

Supplement: Supplementary file 1 — Supplementary Material 1: Fig. S1. a Quantitation of relative protein and mRNA levels of TRIM28. HT1376- XAF1 −/− cells were transfected with an increasing dose of XAF1 as indicated. Expression levels of TRIM28 protein and mRNA levels were determined at 48 h after transfection. Data represent the mean ± SD of triplicate assays. ** P < 0.01 (Student t -test). b cBioportal database analysis showing the inverse correlation of XAF1 mRNA and TRIM28 protein levels in 82 human lung carcinoma tissues. TRIM28 protein and XAF1 mRNA expression were measured by proteomics and transcriptomics analyses, respectively. r 2 , Pearson’s correlation coefficient. Fig. S2. a Comparison of XAF1 effect on etoposide-induced apoptosis in TRIM28 +/+ and TRIM28 −/− sublines of DU145 (Tet-XAF1) cells. XAF1 was induced by addition of tetracycline at 6 h before etoposide (25 μM) treatment. Apoptosis was determined by flow cytometric measurement of sub-G1 fraction and IB assay of cleaved PARP expression at 24 h after etoposide treatment. Data represent the mean ± SD of triplicate assays. Statistical analysis was conducted using Student ‘s t-test, as significance determined as ** P < 0.01. b Comparison of XAF1 effect on TMZ-mediated activation of AMPK phosphorylation and expression of cleaved CASP3 in TRIM28 +/+ and TRIM28 −/− sublines of DU145 (Tet-XAF1) cells. XAF1 was induced by addition of tetracycline at 6 h before TMZ (100 μM) treatment. Fig. S3. a, b Quantitation of p53, AMPKα, H3K9me3, and TRIM28 protein levels. HCT116- TRIM28 +/+ and HCT116- TRIM28 −/− cells were transfected with an increasing dose of XAF1 as indicated. After 48 h transfection, protein levels were determined. Data represent the mean ± SD of triplicate assays. ** P < 0.01 (Student t -test). c, d IB assays for effect of TRIM28 transfection and depletion on expression of TWIST and RLIM in TRIM28 −/− and TRIM28 +/+ sublines of HCT116. e IP assay for comparison of XAF1 effect on MDM2-RLIM interaction in TRIM28 −/− and TRIM28 + [file 43556_2024_224_MOESM1_ESM.pdf]

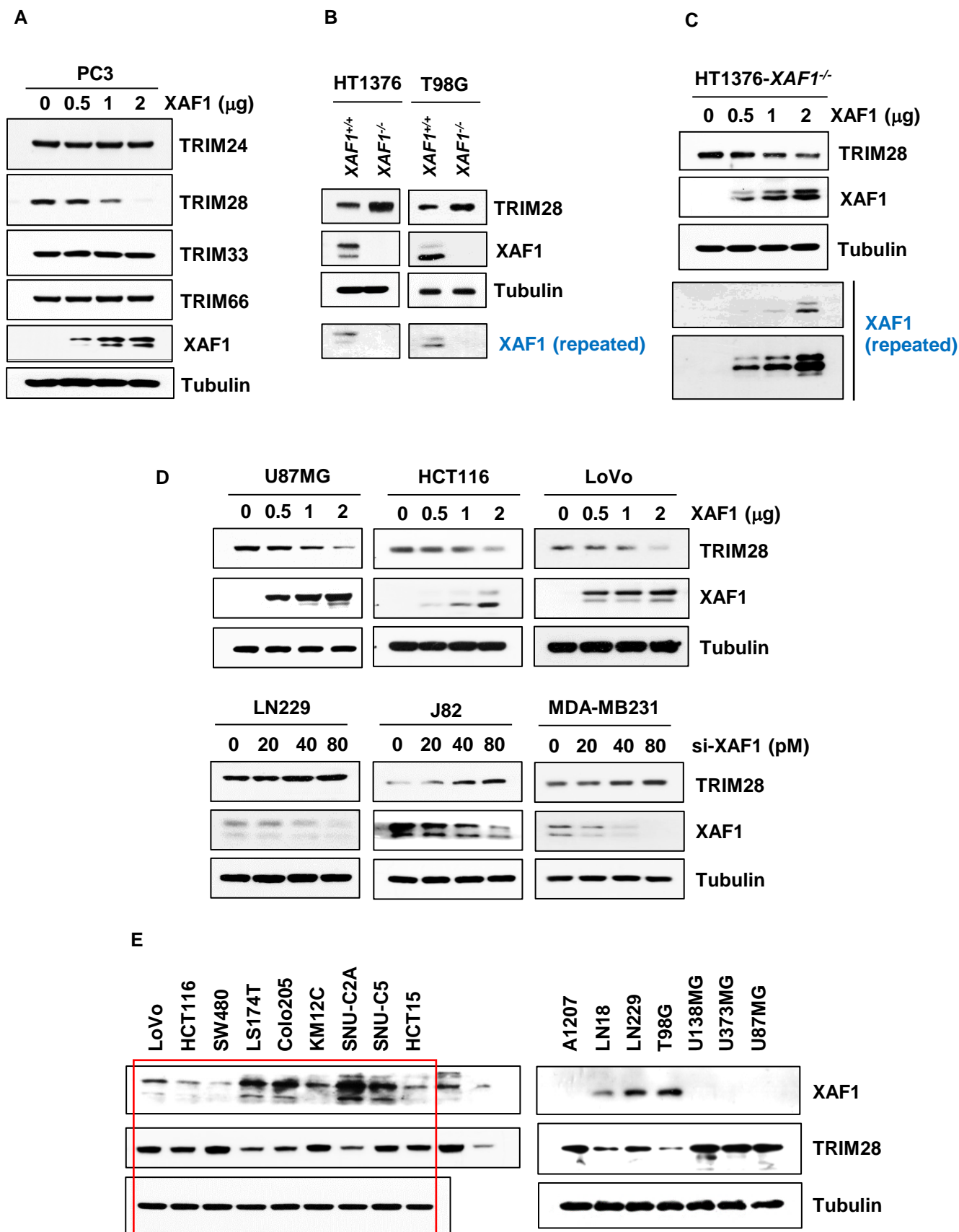

G

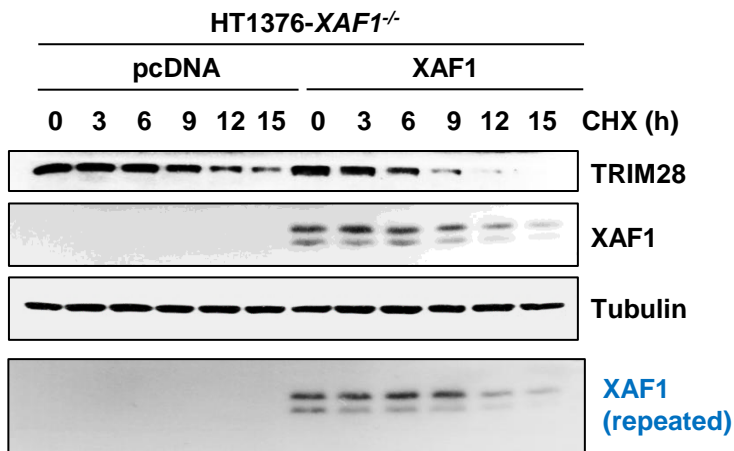

I

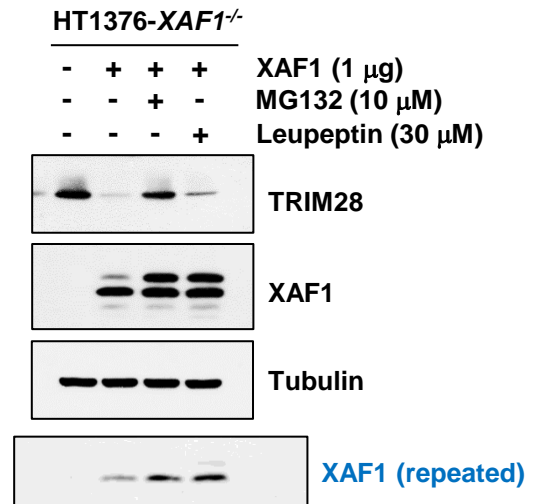

J

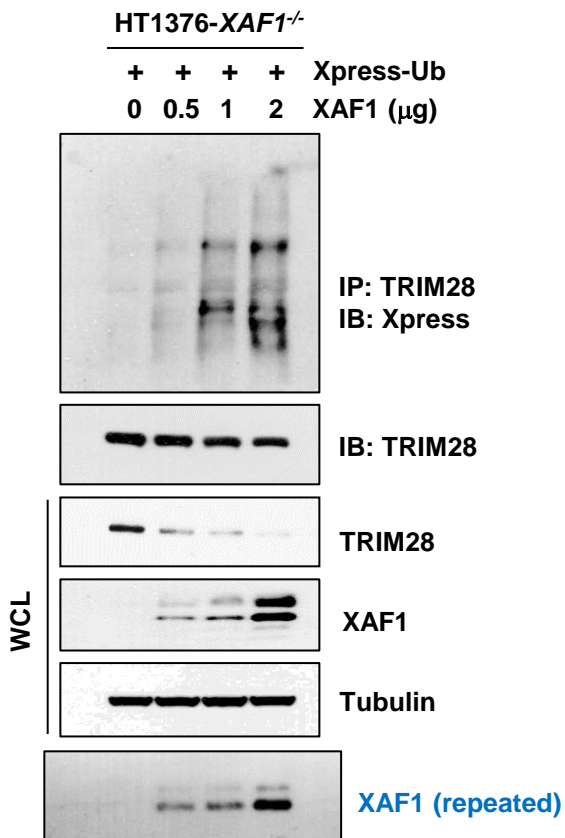

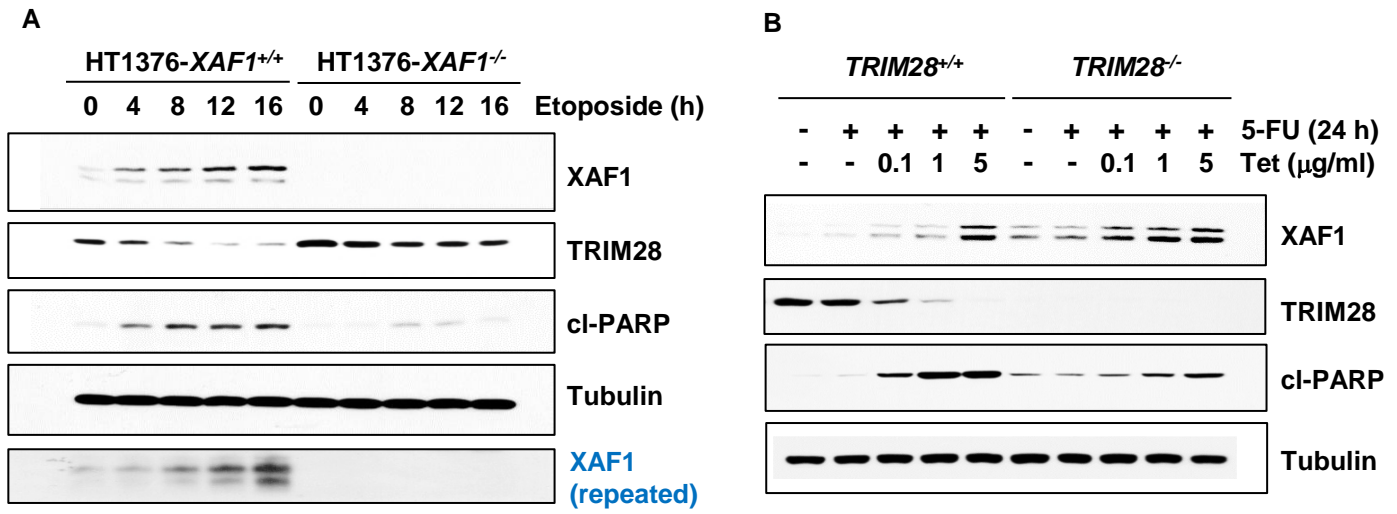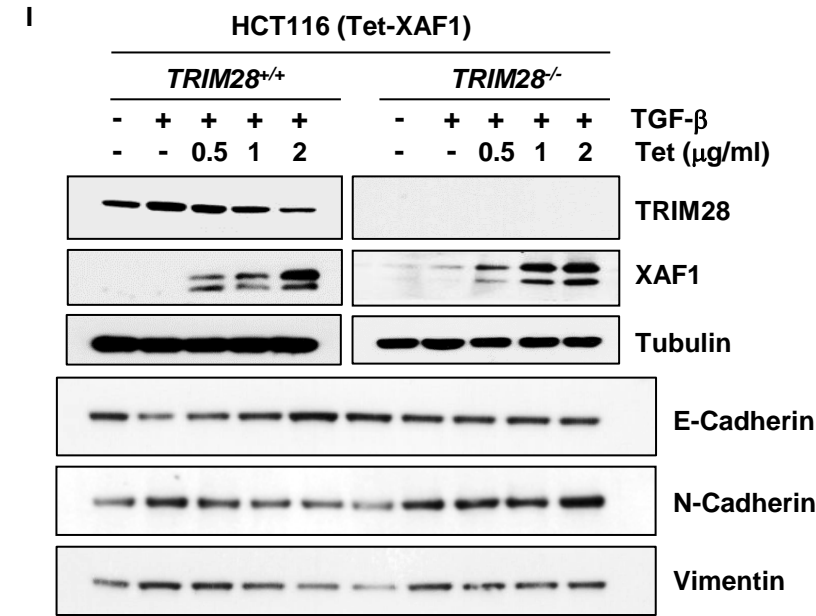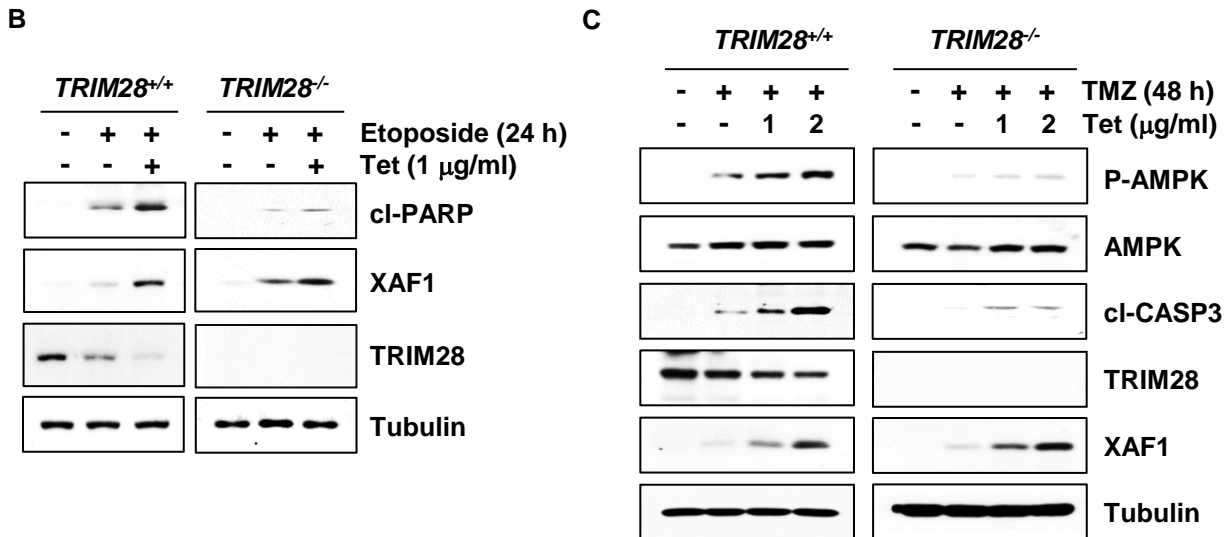

A

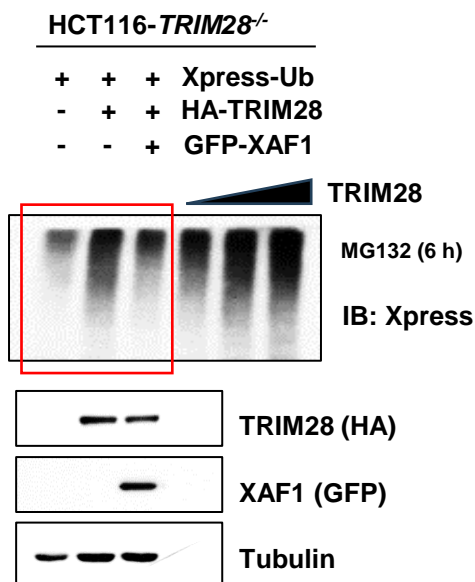

B

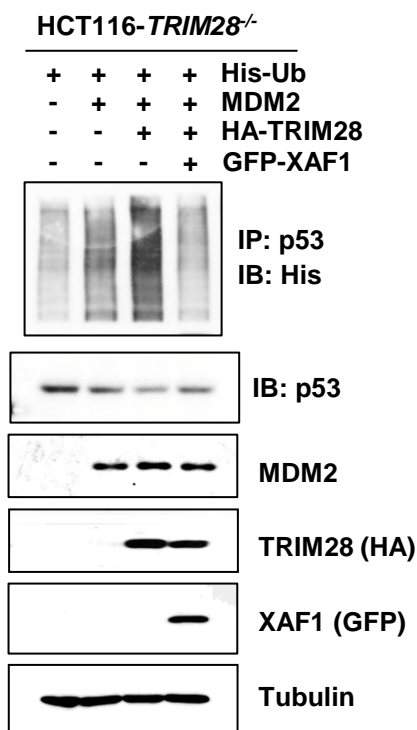

C

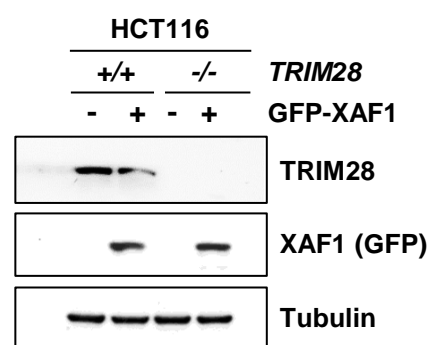

D

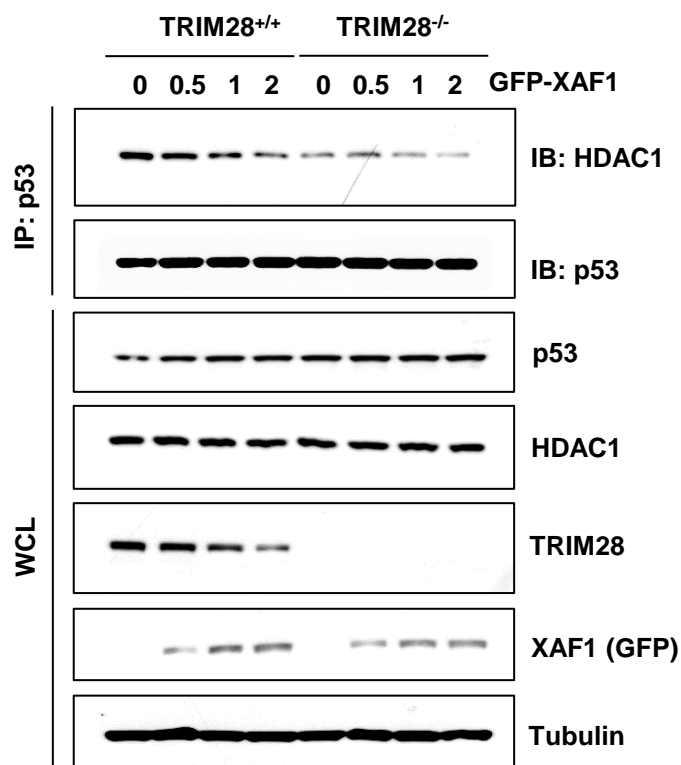

E

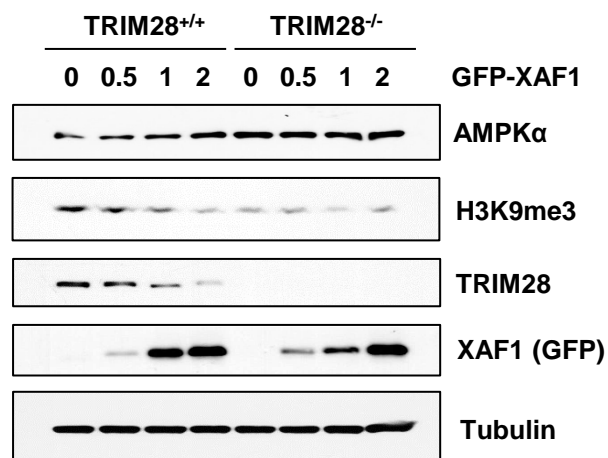

F

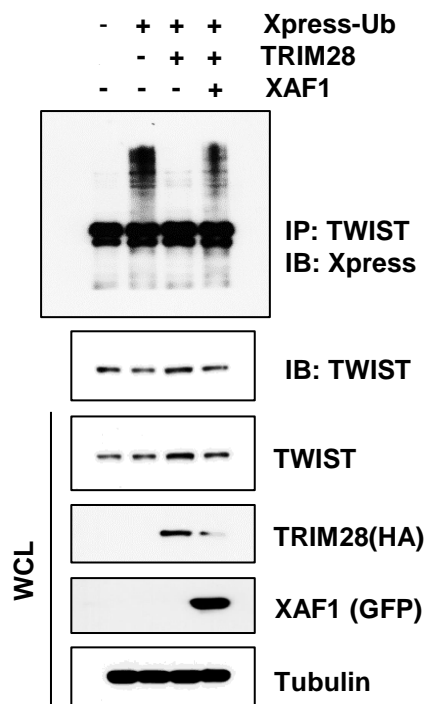

G

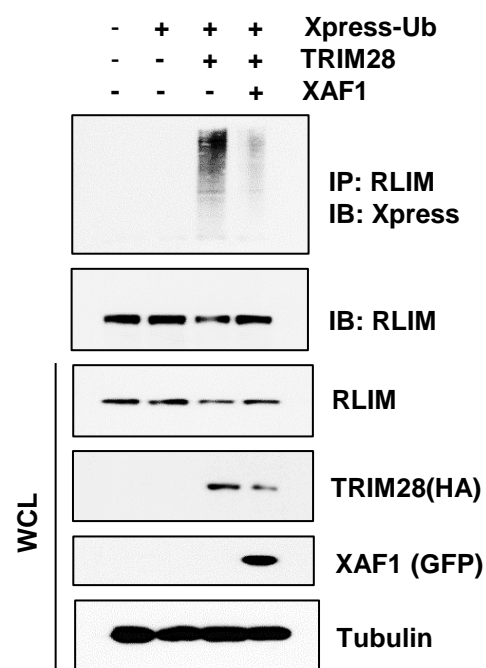

SF3

C

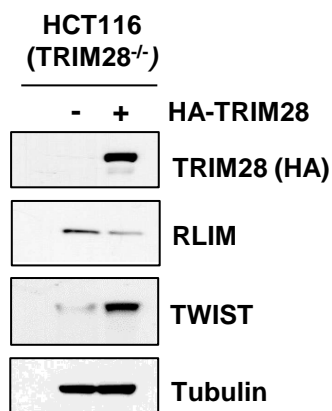

D

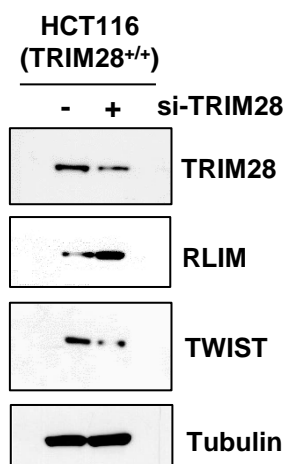

E

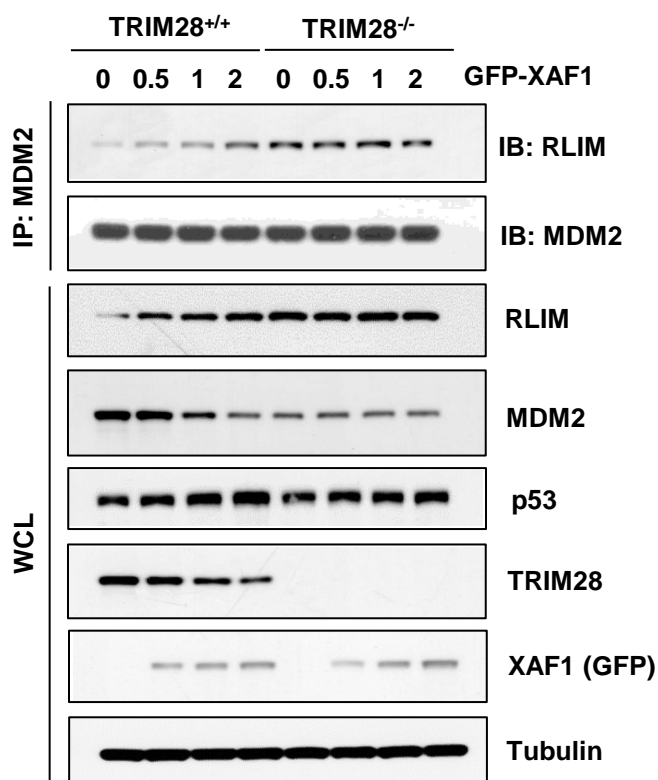

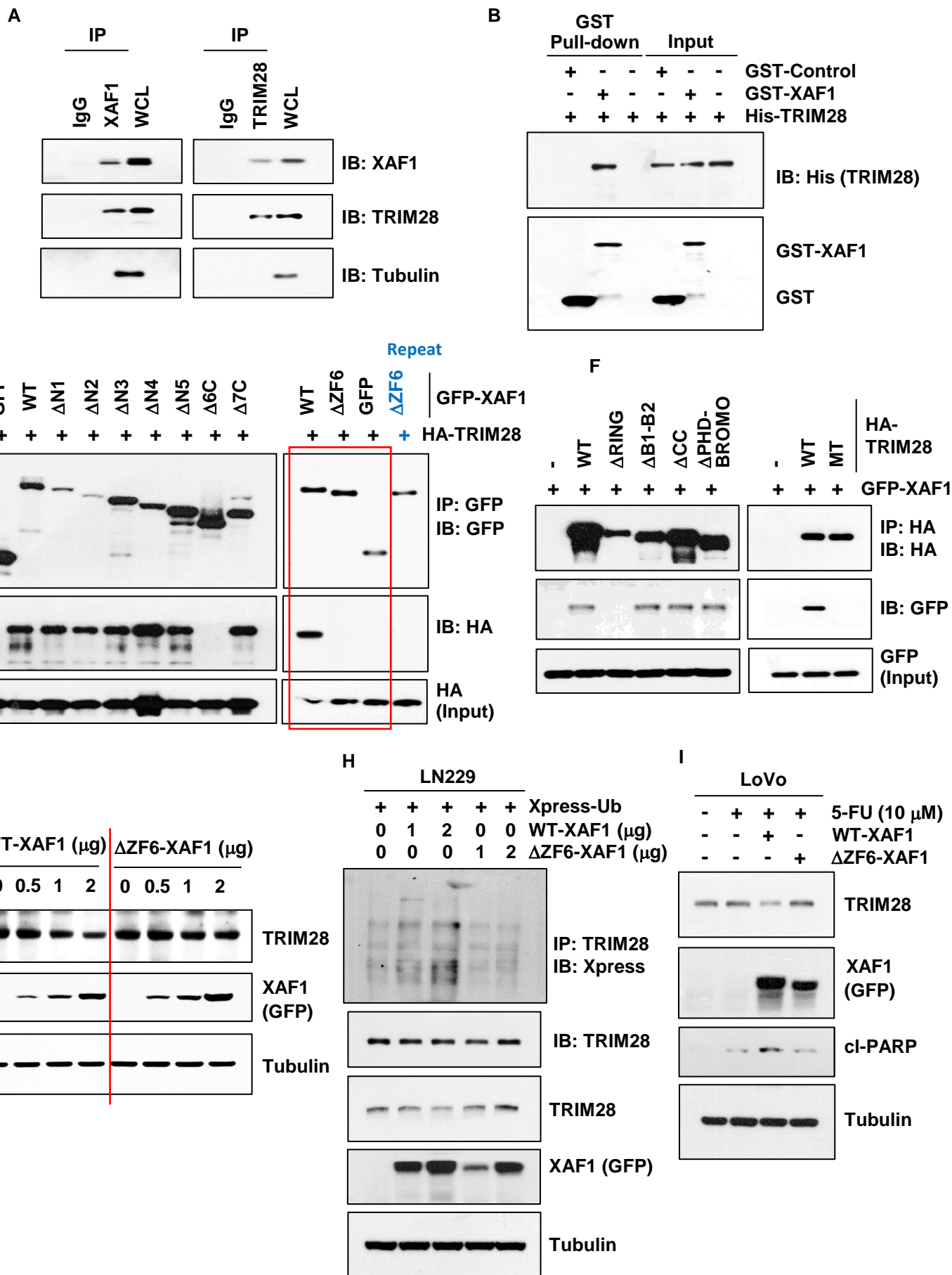

A

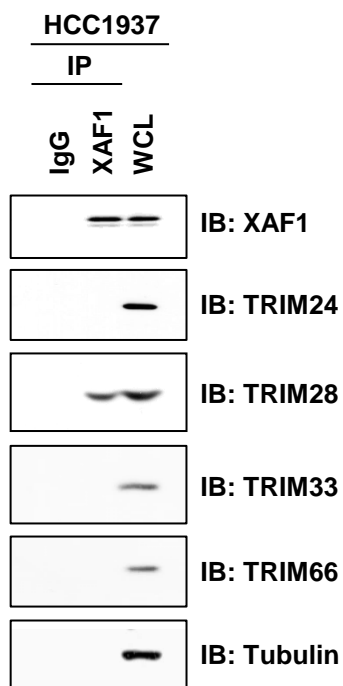

B

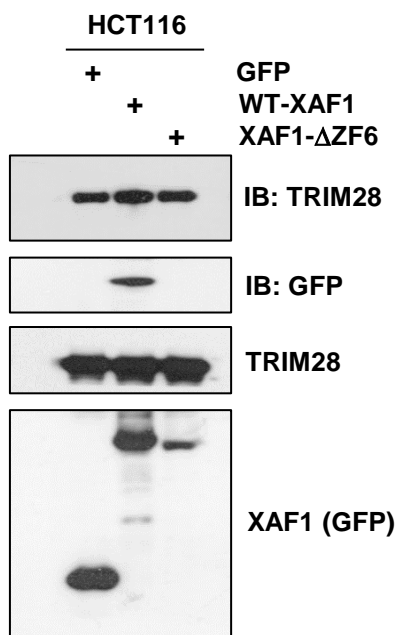

C

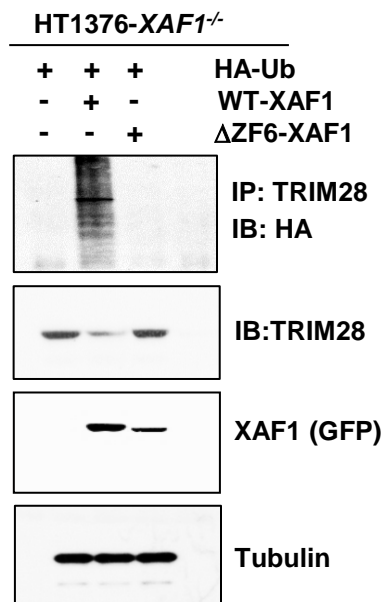

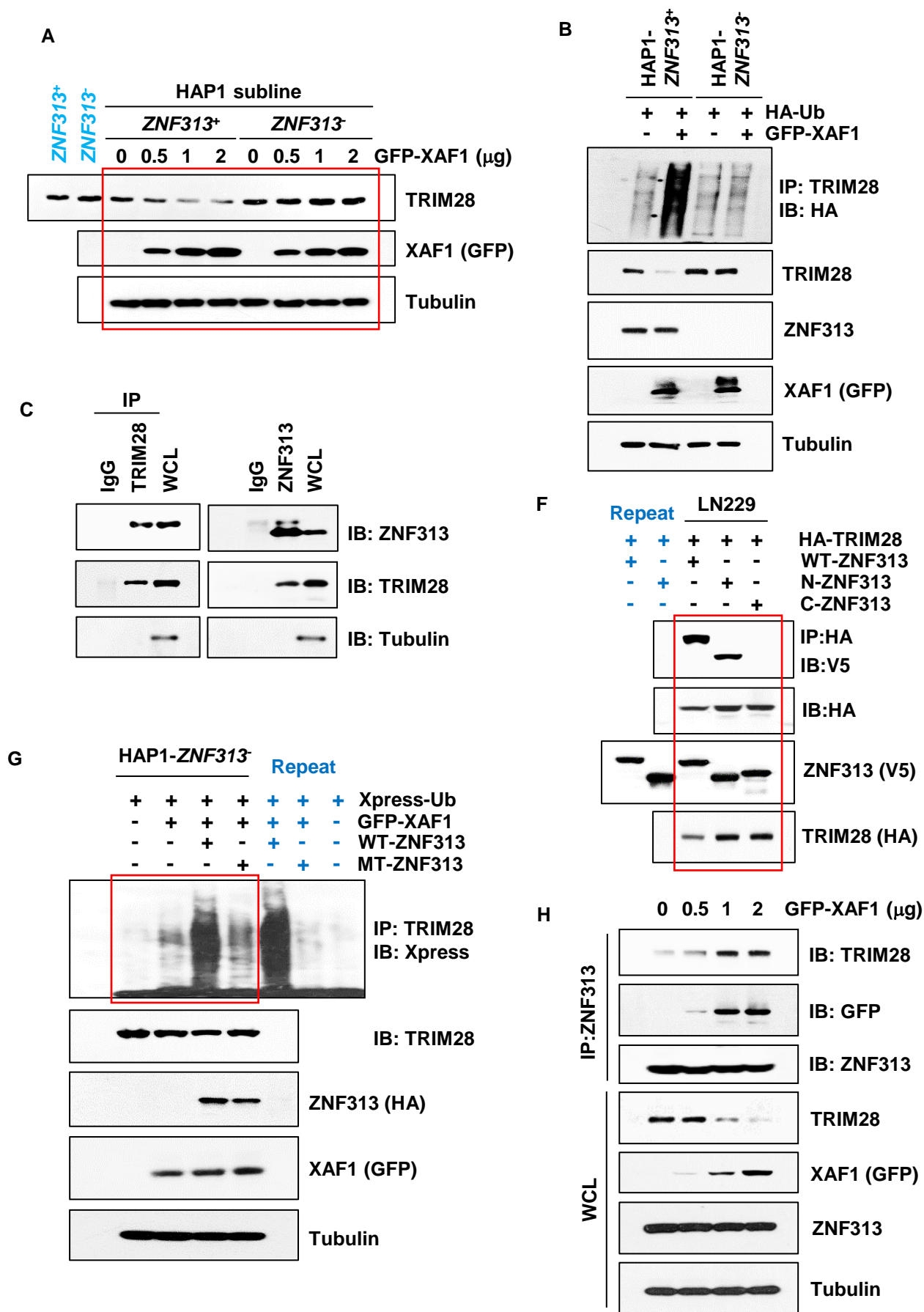

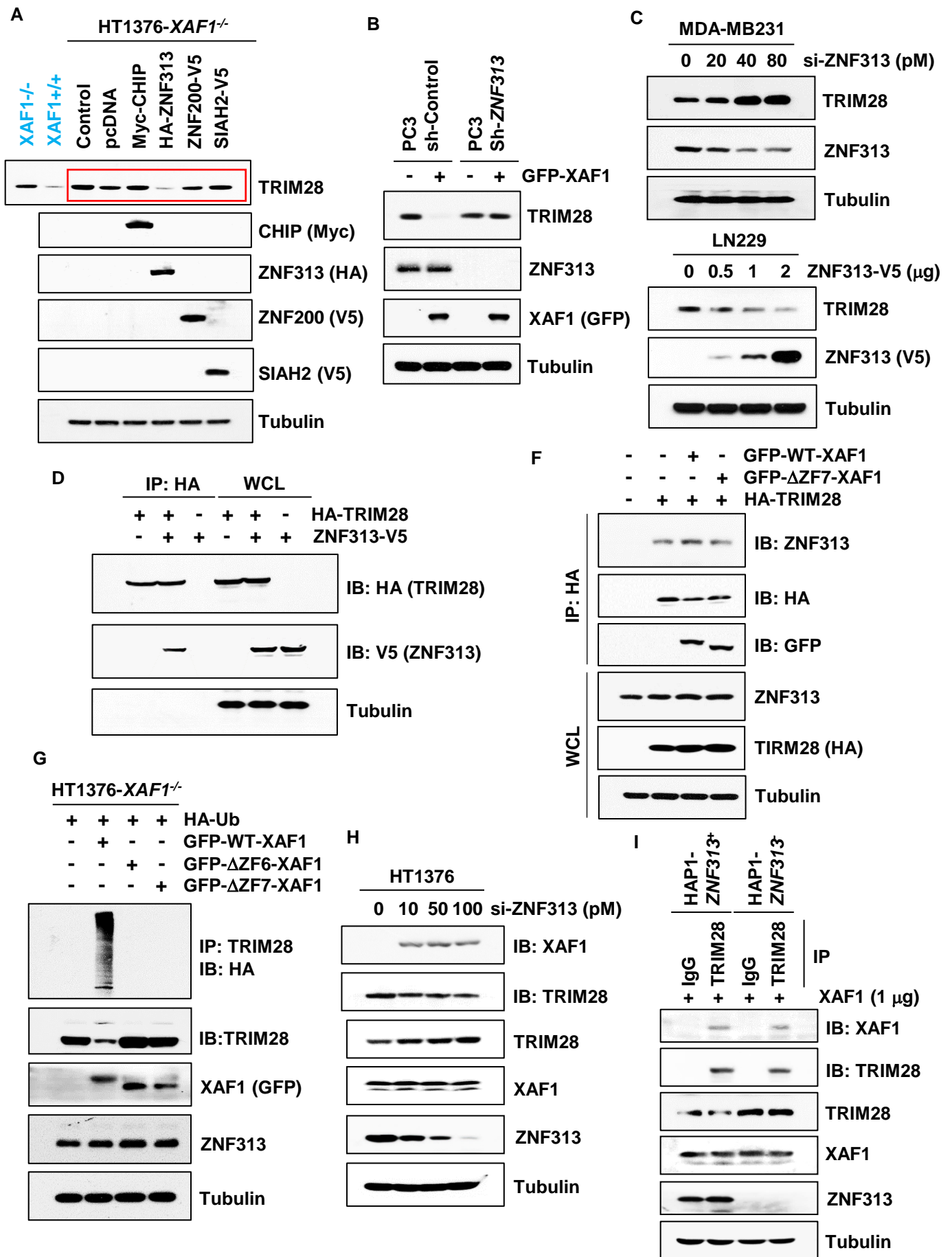

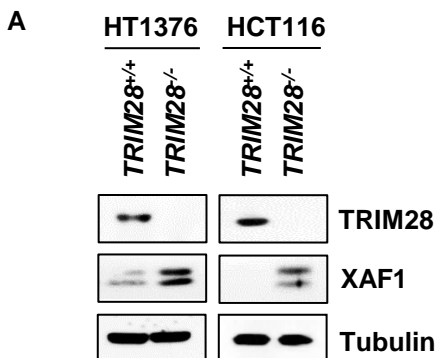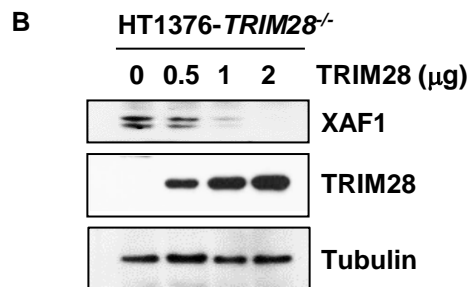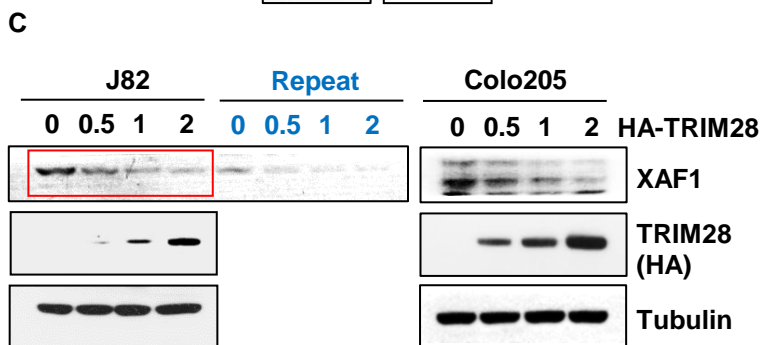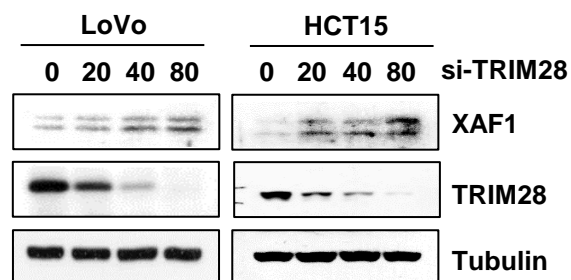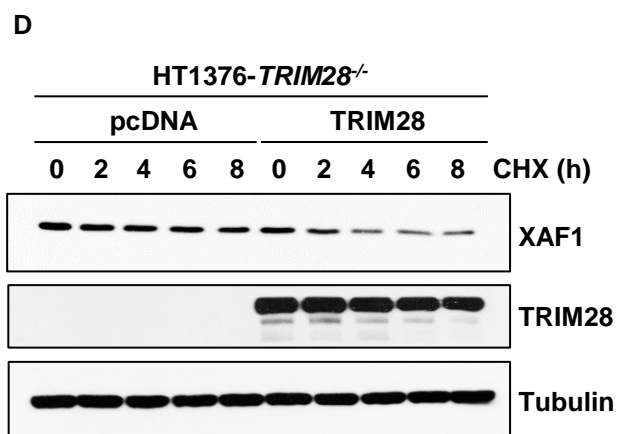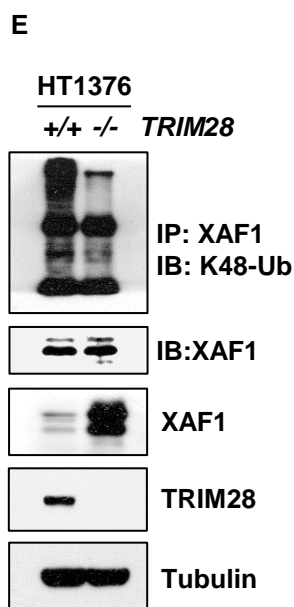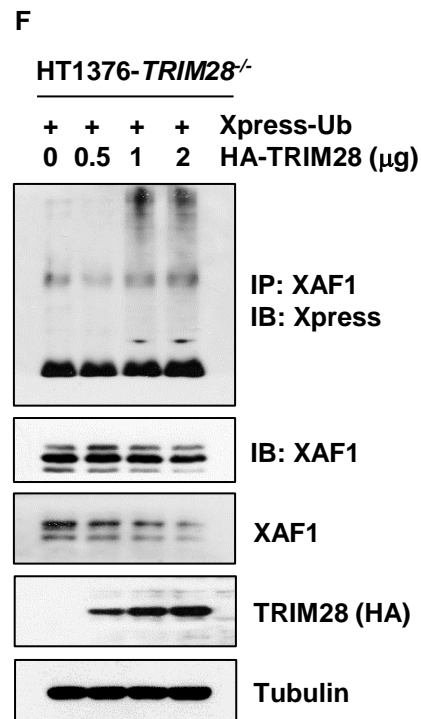

G

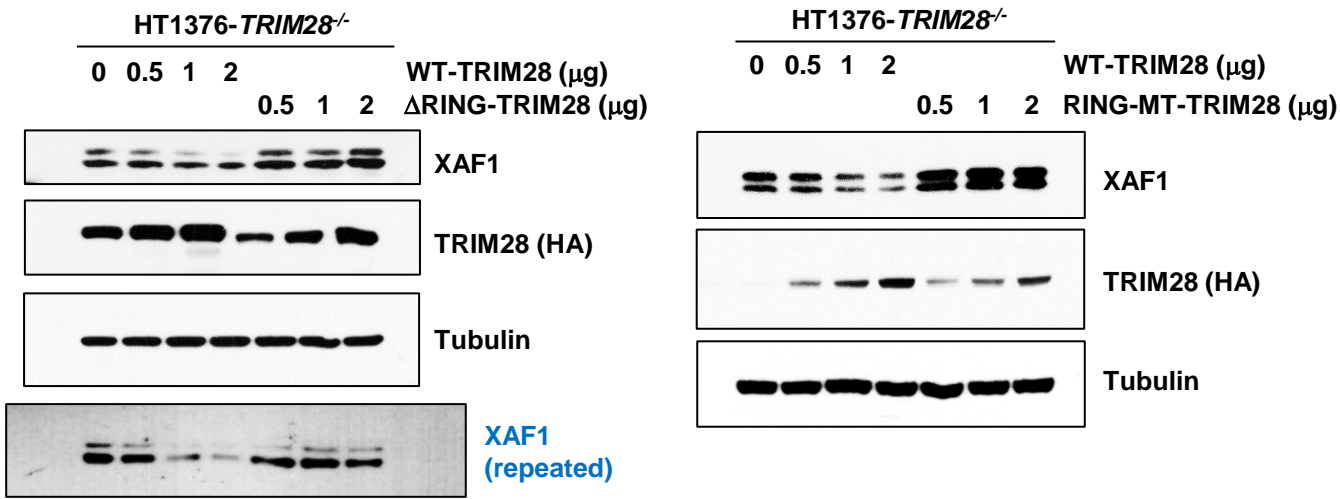

H

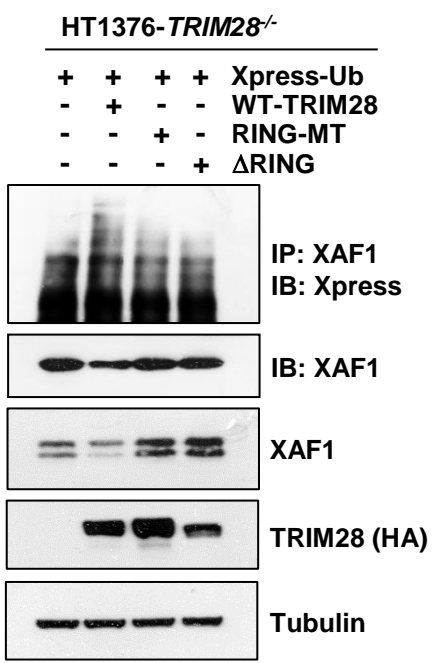

I

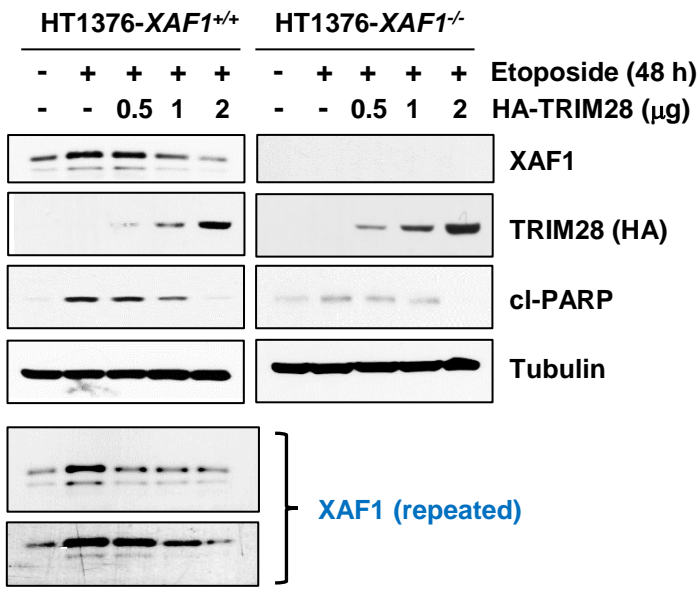

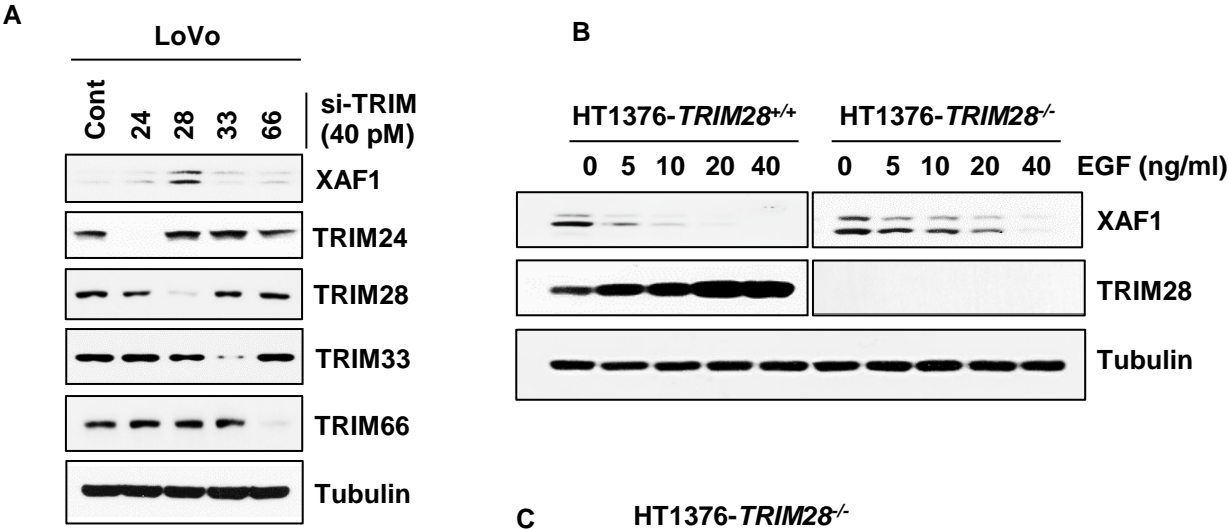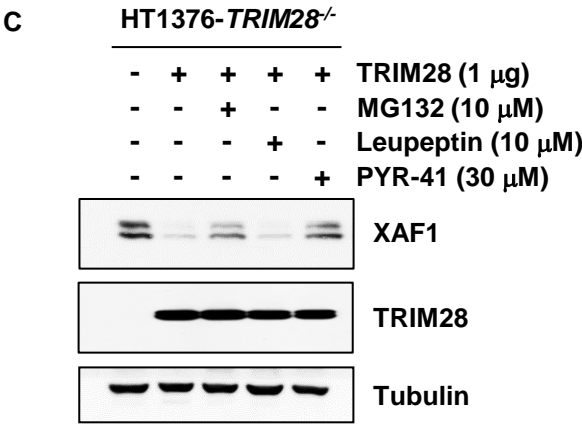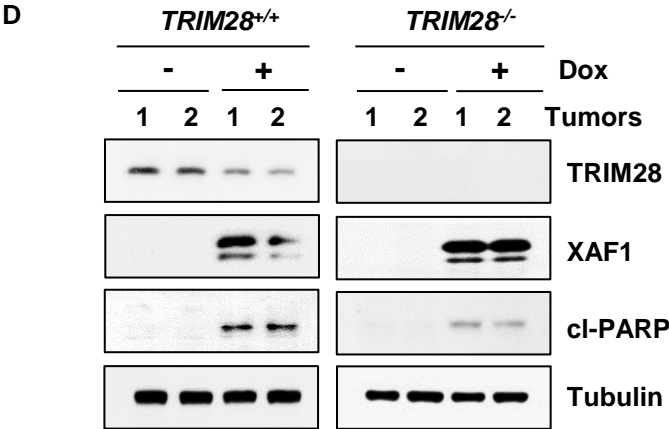

Supplement: Supplementary file 2 — Supplementary Material 2. [file 43556_2024_224_MOESM2_ESM.pdf]
